# Transglutaminase Type 2-MITF axis regulates phenotype switching in skin cutaneous melanoma

**DOI:** 10.1038/s41419-023-06223-y

**Published:** 2023-10-28

**Authors:** Silvia Muccioli, Valentina Brillo, Tatiana Varanita, Federica Rossin, Elisabetta Zaltron, Angelo Velle, Giorgia Alessio, Beatrice Angi, Filippo Severin, Anna Tosi, Manuela D’Eletto, Luca Occhigrossi, Laura Falasca, Vanessa Checchetto, Roberto Ciaccio, Amelia Fascì, Leonardo Chieregato, Ana Paula Rebelo, Marta Giacomello, Antonio Rosato, Ildikò Szabò, Chiara Romualdi, Mauro Piacentini, Luigi Leanza

**Affiliations:** 1 Department of Biology, University of Padua, Padua, Italy; 2 Department of Biology, University of Rome ‘Tor Vergata’, Rome, Italy; 3 Immunology and Molecular Oncology Diagnostics, Veneto Institute of Oncology IOV-IRCCS, Padua, Italy; 4 National Institute for Infectious Diseases IRCCS “Lazzaro Spallanzani”, Rome, Italy; 5 Department of Surgery, Oncology and Gastroenterology (DiSCOG), University of Padua, Padua, Italy; 6 Present Address: Laboratory of Translational Research, Azienda USL - IRCCS di Reggio Emilia, Reggio Emilia, Italy

**Keywords:** Melanoma, Metastasis

## Abstract

Skin cutaneous melanoma (SKCM) is the deadliest form of skin cancer due to its high heterogeneity that drives tumor aggressiveness. Melanoma plasticity consists of two distinct phenotypic states that co-exist in the tumor niche, the proliferative and the invasive, respectively associated with a high and low expression of MITF, the master regulator of melanocyte lineage. However, despite efforts, melanoma research is still far from exhaustively dissecting this phenomenon. Here, we discovered a key function of Transglutaminase Type-2 (TG2) in regulating melanogenesis by modulating MITF transcription factor expression and its transcriptional activity. Importantly, we demonstrated that TG2 expression affects melanoma invasiveness, highlighting its positive value in SKCM. These results suggest that TG2 may have implications in the regulation of the phenotype switching by promoting melanoma differentiation and impairing its metastatic potential. Our findings offer potential perspectives to unravel melanoma vulnerabilities via tuning intra-tumor heterogeneity.

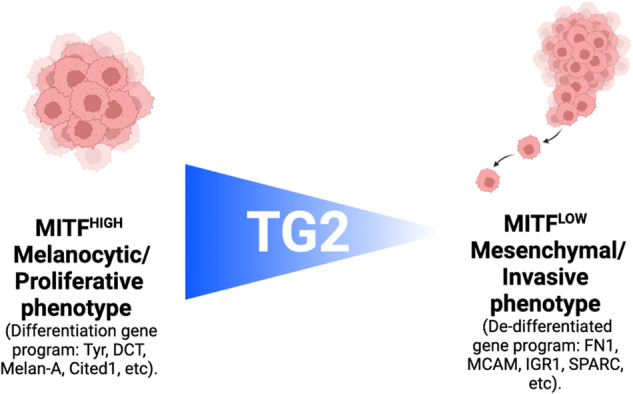

## Introduction

Melanoma is the deadliest sub-type of skin cancer, mainly due to its high metastatic potential. Although immunotherapies and MAP-kinases targeted drugs have widened the treatment options of metastatic patients, the development of resistance and tumor recurrence is still limiting the clinical benefits of these novel approaches [1]. The main process that influences the ability to evade therapeutic treatments and fosters tumor aggressiveness is melanoma intra-tumor heterogeneity [2]. Based on gene signature profiling, melanoma has been divided into two prevalent phenotypically distinct sub-populations of cells that co-exist in bulk tumor tissues: the proliferative and the invasive states [3–6]. The proliferative motif is characterized by rapidly proliferating cells and high expression of the microphthalmia-associated transcription factor (MITF), the master regulator of pigmentation. Conversely, a melanoma cell needs to push invasiveness at the expense of proliferation to drive metastasis formation, increasing cellular plasticity and stem-like features. These signatures distinguish the invasive state, which is associated with low expression of MITF [1, 7–10]. MITF has been extensively studied in melanoma cells where its transcriptional activity modulates target genes involved in pigmentation, such as *TYR*, *MC1R*, *DCT*, and *MLANA* [11]. Noticeably, MITF is not only relevant for melanogenesis, as it exerts numerous functions in melanoma cell homeostasis by modulating proliferation, migration, immunosuppression, and many other cancer hallmarks [12]. The balancing of MITF expression is complex and tightly restrained, exhibiting both transcriptional and post-translational control [13]. In this setting, MITF’s upstream regulators (SOX10, PAX3, EDNRB and CREB) are known as drivers of the invasive to proliferative switch, while MITF’s downstream targets (MLANA, PMEL, DCT, TYRP) work as markers of the proliferative signature [14].

The high mutational burden of melanoma distinguishes this type of cancer as one of the most immunogenic, making it a suitable target for anti-cancer immunotherapy. However, immunotherapy-based approaches represent a relatively late discovery for melanoma. Nevertheless, a significantly higher success rate of this type of treatment in combination with chemotherapy, radiotherapy, or targeted molecular therapy has been observed, even if it is associated to a panel of side effects that can affect one or more organs and may limit its use [15].

Transglutaminase type-2 (TG2) is the most well characterized and studied member of a family of eight isoenzymes (TG1-7 and coagulation factor XIII) that catalyse the crosslinking between Glutamine and Lysine residues on peptides or proteins. TG2 is ubiquitously expressed and its localization within cells includes all cellular districts, being in the nucleus, cytosol, mitochondria, endoplasmic reticulum, and extracellular environment [16]. However, beside this primary function, TG2 also displays a wide variety of different activities, like deamidase, GTPase, isopeptidase, adapter/scaffold, protein disulfide isomerase, kinase, hipusination regulation, serotonilation activities [17]. Thanks to its multifunctionality, TG2 is involved in many cellular processes, like cell growth and differentiation, cell death, autophagy, inflammation, macrophage’s phagocytosis, tissue repair, fibrosis and wound healing, ECM assembly, and remodelling [18]. TG2 level of expression is sensitive to changes in physiological conditions, since the *TGM2* gene (which encodes for TG2), is regulated by several agents and stimuli, like apoptotic signals, viral infections, ER stress, hypoxia, inflammation, and cancer-activated pathways. As several molecules impact on *TGM2* activation, many signalling pathways are involved in its regulation and consequent behaviour. For instance, the *TGM2* promoter region contains various responsive elements able to induce or inhibit TG2 expression during inflammation and hypoxia, two key oncogenic processes characterizing the tumour microenvironment (TME) [19]. However, the role of TG2 in cancer is still controversial and far to be fully elucidated, since it has been reported as both a potential tumour-suppressor and a tumour-promoting factor [20]. Certainly, the function of TG2 is tissue-specific [20]: in particular, we recently demonstrated by analysing public transcriptomic databases a correlation between TG2 expression, good SKCM overall survival, and a positive regulation of immune response in SKCM [21]. These results indicate that TG2 expression might serve as a good prognostic factor in patients with SKCM, as well as a biomarker for the therapeutic strategy to be adopted [21].

In the present work, we gained new insight into the function of TG2 in cutaneous melanoma. We discovered that TG2 expression is required during the process of melanoma pigmentation by modulating MITF expression and activity. In turn, we have shown that TG2 expression is also related to a reduced capacity of melanoma cells to form metastasis both in vitro and in vivo, highlighting that TG2 is involved in melanoma invasion and could be associated to the phenotype switching of melanoma cells. These findings could help to better understand the intratumor variability of cutaneous melanoma, unveiling novel vulnerabilities and offering unexplored treatment perspectives for the cure and improvement of the prognosis of metastatic patients.

## Results

### TG2 positively correlates with better prognosis in SKCM patients only

**Fig. 1:**
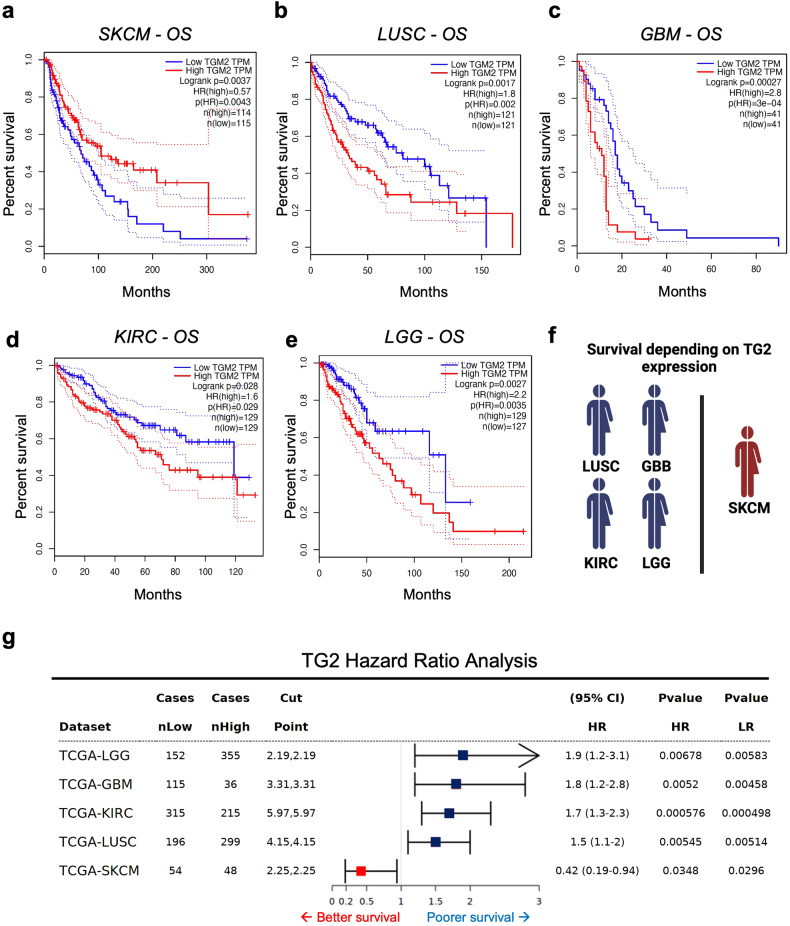
Analysis of the TG2 significant clinical value in TCGA cancer datasets. **a–e** Overall survival based on TG2 expression level in SKCM (Skin cutaneous melanoma), LUSC (Lung squamous cell carcinoma), GBM (Glioblastoma multiforme), KIRC (Kidney renal clear cell carcinoma), and LGG (Brain lower grade glioma) was obtained through Kaplan-Meier analysis by sorting samples for high and low TG2 expression groups according to the quartile (Cutoff-High=25%; Cutoff-Low=75%) on GEPIA. Percent survival was plotted, and *p*-values were shown as per figure specification, respectively. **f** Schematic representation of the impact of TG2 expression on LUSC, GBM, KIRC, LGG, and SKCM. TG2 expression is a worst prognostic signature in LUSC, GBM, KIRC, and LGG (represented in blue), whereas it has a positive clinical value in SKCM only (in red). **g** Forest plot showing the detailed table of the Univariate Cox-Regression Survival Analysis of TG2 expression in LGG, LUSC, GBM, KIRC, and SKCM, retrieved using the Survival Genie Software. The plot shows the hazard ratio and 95% confidence intervals associated with the two considered groups of patients (high and low expression of TG2), along with Walt test and log-rank *p*-values. Cut-off values applied to the two subsets of patients and the sample number in each group are also shown. To assess the Hazard Ratio (HR) based on TG2 expression in LGG, GBM, KIRC, LUSC, and SKCM primary tumors, we used the Cutp option for the Cut-off establishment (the cut-point is estimated based on martingale residuals using the survMisc package to stratify patients into high and low groups). Squares represent the Hazard Ratio (HR), while the horizontal lines depict the upper and lower limits of the HR 95% confidence interval (arrow pointing to the upper limit indicates that the interval is higher than the maximum shown). Confidence Interval (CI). Likelihood Ratio (LR). Negative significant prognostic values are represented in blue squares, while positive associations in red.

Transglutaminase Type-2 (TG2) is a multifunctional enzyme that is reported to be involved in all the stages of carcinogenesis. Its impact on oncogenic processes is still highly debated, as it has been described both as a tumor-promoting and a suppressor factor [20]. To fill this knowledge gap, we analyzed the effect of TG2 expression in 32 histotypes of tumors on the TCGA dataset by taking advantage of the GEPIA software [22]. Among all the cancer types, Kaplan–Meier analysis revealed that TG2 expression correlates with patient prognosis in only 5 out of 32 tumors (Fig. 1a–f). In particular, a high TG2 expression is correlated with a poorer survival rate in LUSC (Lung squamous cell carcinoma) (*p* = 0.002) (Fig. 1b), GBM (Glioblastoma multiforme) (*p* = 0.0027) (Fig. 1c), KIRC (Kidney renal clear cell carcinoma) (*p* = 0.029) (Fig. 1d), and LGG (Brain Lower Grade Glioma) (*p* = 0.0035) (Fig. 1e). These data are in agreement with previous literature [23–26]. Interestingly, the only tumor type in which TG2 has a positive value on Overall Survival is SKCM (Skin Cutaneous Melanoma) (*p* = 0.0043) (Fig. 1a). Hazard Ratio analyses obtained using the Survival Genie software [27] confirmed that TG2 has a positive prognostic role in SKCM primary tumors only (Fig. 1g). These results are in line with our previous research in which we outlined a novel promising positive clinical value for TG2 in skin cancer [21], though the molecular mechanism behind TG2 function was not fully elucidated.

### TG2 ablation impairs melanoma pigmentation and increases its invasiveness

**Fig. 2:**
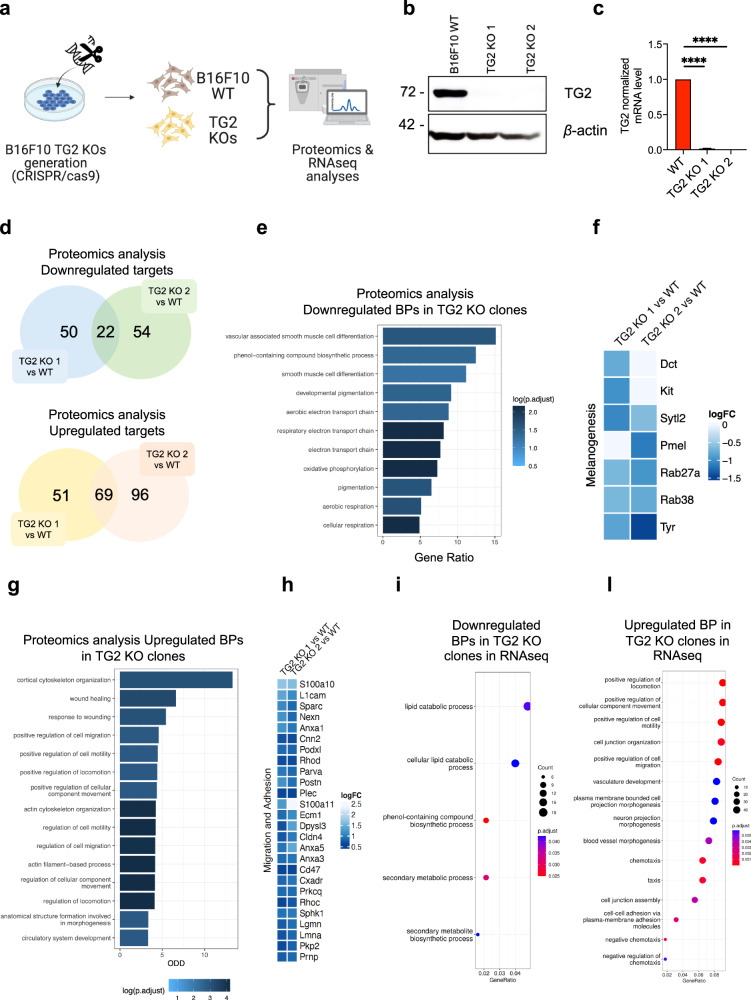
Generation and multi-omics characterization of TG2 knock-out melanoma B16F10 cells. **a** Schematic representation of the employed strategy. B16F10 TG2 KO clones were generated by means of the CRISPR/Cas9 genomic editing tool. After obtaining the clones, they were subjected along with the B16F10 WT cell line to Proteomics profiling and RNA-seq analyses. **b** Immunoblot analyses showing the obtained TG2 KO clones, namely TG2 KO 1 and TG2 KO 2. Actin was used as loading control. **c** *TGM2* expression evaluated by qRT-PCR analysis in B16F10 WT cells and TG2 KO clones (number of independent biological replicates = 8). Statistical significance was calculated with One-Way ANOVA and specified with asterisks (^****^p < 0.0001). Data are represented as mean ± SEM. **d** Venn diagrams showing the differentially expressed proteins from comparative Proteomic analyses of the TG2 KO clones. Comparisons were divided in Up and Down-regulated Proteomics targets (hits and candidate proteins). Areas of overlap indicate shared protein targets. Statistically significant targets were defined based on *adj.p* < 0.05 (adjusted *p* < 0.05). Proteins were annotated as “hits” with FDR < 5% and a fold change of at least 100% and as “candidates” with FDR < 20% and a fold-change of at least 50%. **e** Bar plot representative of the GO enrichment analyses of the top 11 downregulated Biological Processes (BPs). Bar color represents the *adj.p-value* (dark blue= most significant). Bar lengths refer to the proportion of enriched proteins for each term. **f** Heat map of comparative proteomic analysis of the melanogenesis related proteins was generated using pheatmap R package. **g** Bar plot representative of the GO enrichment analyses of the top 15 upregulated Biological Processes (BPs). Bar color represents the *adj.p-value* (dark blue= most significant). Bar lengths refer to the proportion of enriched proteins for each term. **h** Heat map of comparative proteomic analysis of the migration and adhesion proteins was generated using pheatmap R package. Dot plots representative of down (**i**) and up (**l**) regulated GO of Biological Processes analyses performed on significantly differentially expressed genes (DEGs) obtained from RNAseq profiling of the TG2 KO 2 clone (FDR < 0.01). Bubble colors represent the *adj.p-value* (red=most significant). The rich factor refers to the proportion of enriched genes for each term.

We decided to employ the CRISPR/Cas9 technology to generate TG2 knock-out cell lines to consequently identify differentially expressed genes and their molecular pathways that regulate and/or are regulated by TG2 ablation. For this purpose, we took advantage of a multi-omics approach, combining data from Proteomics and RNAseq analyses (Fig. 2a). At first, we generated single clones from a CRISPR/Cas9-mediated TG2 KO B16F10 cell line by adapting the protocol of F. Ann Ran et al., 2013 [28] (Fig. S2a). We used pairs of gRNAs targeting the promoter region of the gene, including the 5’UTR and the transcription initiation site, to prevent both the transcription and translation of TG2 (Fig. S2c). This strategy was adopted as the 3′ TG2 coding region partially overlaps with one of the isoforms of the *RPRDIP* gene, so that the complete deletion of *TGM2* would generate off-target effects (Fig. S2b). Through this procedure, we were able to obtain significant TG2 protein and mRNA KO in two independent clones, named “TG2 KO 1” and “TG2 KO 2”, (Fig. 2b, c), which were also validated by PCR (Fig. S2d, e) and selected for further experiments.

Subsequently, we proceeded with a Mass Spectrometry analysis to determine the differences in the Proteomic profiles of the TG2 KO clones against the wild-type B16F10 line. Proteins classified as “hits” (FDR < 5%, Fold Change at least 100%) and “candidates” (FDR < 20%, Fold Change at least 50%) were taken into consideration. Overall, we found 50 proteins whose expression was downregulated in the TG2 KO 1, 54 in the TG2 KO 2, and 22 downregulated proteins common to both clones. Concerning the upregulated targets, 51 proteins were found in TG2 KO 1, 96 in TG2 KO 2, and 69 in common (Fig. 2d). A comprehensive list of both up and downregulated Proteomic targets is reported in Supplementary Tables 1–4.

To infer the biological significance of TG2 in our model, we performed Gene Ontology (GO) enrichment analysis on the list of the identified Proteomics targets. In Figs. 2e, g we report the first 15 statistically downregulated (Fig. 2e) and upregulated (Fig. 2g) Biological Processes categories in TG2 KO clones. In particular, by taking into consideration the union of the downregulated targets in both clones, we found that the main affected Biological Processes in the TG2 KO cell lines are linked to the process of melanogenesis (Fig. 1e-S3a). Interactome analyses conducted on the downregulated targets with STRING and clustered using ClusterONE Software on Cytoscape demonstrated that several targets are relevant for the differentiation of melanocytes and endocytosis, like Kit [29], Dct [30], Tyr [31], Pmel [32], Rab27a [33], Rab38 [34], and Sytl2 [35] (Fig. 2f-S3b). These targets are mainly distributed in melanosomes (the cellular organelles responsible for the synthesis of melanin [36] and their membranes, in the pigment granules, and in the vesicle systems that are necessary for the transport of melanin, as reported in the GOs of Cellular Components (CC) (Fig. S3c). Interestingly, alterations in these proteins are found in Hair Hypopigmentation (HP) (Fig. S3c). On the other hand, considering the Biological Processes associated with the upregulated targets, numerous categories referring to chemotaxis, cell adhesion, and remodeling of the cytoskeleton that supports cell movement have been identified (Fig. 2g). Reflecting their function, these proteins are mainly localized at the level of cell junctions, on cell protrusions, in the cytoskeleton, and in focal adhesions (Fig. S3e).

To further investigate the role of TG2 in melanoma, we decided to accompany the Proteomics data that we obtained also with an RNAseq and Transcriptomic profiling of our model. Considering the similarity in the Proteomic profiles of the two TG2 KO clones, we decided to conduct the RNAseq analysis only on the TG2 KO 2 clone with respect to the WT. According to the Proteomics, RNAseq analyses revealed among the significantly downregulated GO-terms the “*phenol-containing compound biosynthetic process*”, “*secondary metabolic processes*”, and “*secondary metabolite biosynthetic process*” (Fig. 2i). These categories include transcripts whose expression is necessary for the pigmentation process, such as Cited1 [13], Snca [37], Slc24a5 [38], and the aforementioned Tyr and Pmel (Fig. S3d). At the same time, the most upregulated categories of transcripts in the TG2 KO are involved with positive regulation of chemotaxis and cell junction regulation (Fig. 2l). Among them, we found Sparc [39], Cd47 [40], Plec [41], Anxa1 [42], Anxa 3 [43], and Anxa 5 [44] (Fig. S3f–h), genes strongly involved in the metastatic processes of melanoma. A large part of these up or downregulated transcripts are present among the targets identified in Proteomics of both KO clones (Tables 1–4). As a further confirmation, qRT-PCR analyses on some transcripts identified as differently expressed with respect to the WT line were validated on both KO clones (Fig. S4a–f).

**Table 1:** sgRNAs used to generate TG2 B16F20 KO clones by CRISPR/Cas9.

TGM2_gRNA1	GGCTATAAGTTCGCGCCGCG
TGM2_gRNA2	CGCTCCAGGTGTCTGTTCCG
TGM2_gRNA3	AGAATTAACGGCTATGCCCT

**Table 2:** PCR primers used for KO validations on genomic DNA after B16F10 clonal selection.

FW_TGM2_screen_1	GAGGGTTATATGTGGCTTCGTGGA
RV_TGM2_screen_1	ACCTTATAGTCAGCTGGGATTCTGG

**Table 3:** qRT-PCR primers for KO validations.

mm_Actin_FOR	AGTGTGACGTTGACARCCGT
mm_Actin_REV	TGCTAGGAGCCAGAGCCGTA
mm_TGM2_FOR	GGAGGAGCGACGGGAATATG
mm_TGM2_REV	ATTCCATCCTCGAACTGCCC

**Table 4:** Antibodies used for Western Blot protein bands detection.

ANTIBODY	SOURCE	IDENTIFIER	DILUTION
β-actin	Millipore	Cat# mab 1501	1:6000
β-catenin	Sigma-Aldrich	Cat# C7082	1:1000
DCT	Santa Cruz Biotechnology	sc-74439	1:500
Fibronectin 1	Sigma-Aldrich	Cat# F3648	1:1000
Lamin A/C	Santa Cruz Biotechnology	sc-6215	1:1000
MAPK (ERK1/2)	Cell signaling Technology	Cat# 4695	1:1000
Melan-A	Cell signaling Technology	Cat# 64718	1:1000
MITF	Invitrogen	Cat# MA5-14154	1:1000
p38	Cell signaling Technology	Cat# 9212	1:1000
TG2	Cell signaling Technology	Cat# 3557	1:1000
Tyrosinase	Santa Cruz Biotechnology	Sc- 20035	1:1000
Vinculin	Millipore	Cat# mab 4505	1:6000

These results suggest that TG2 can modulate targets involved in pigmentation and migration capacity of melanoma cells, two key processes for melanoma plasticity.

### Lack of TG2 affects melanin synthesis in vitro and in vivo

**Fig. 3:**
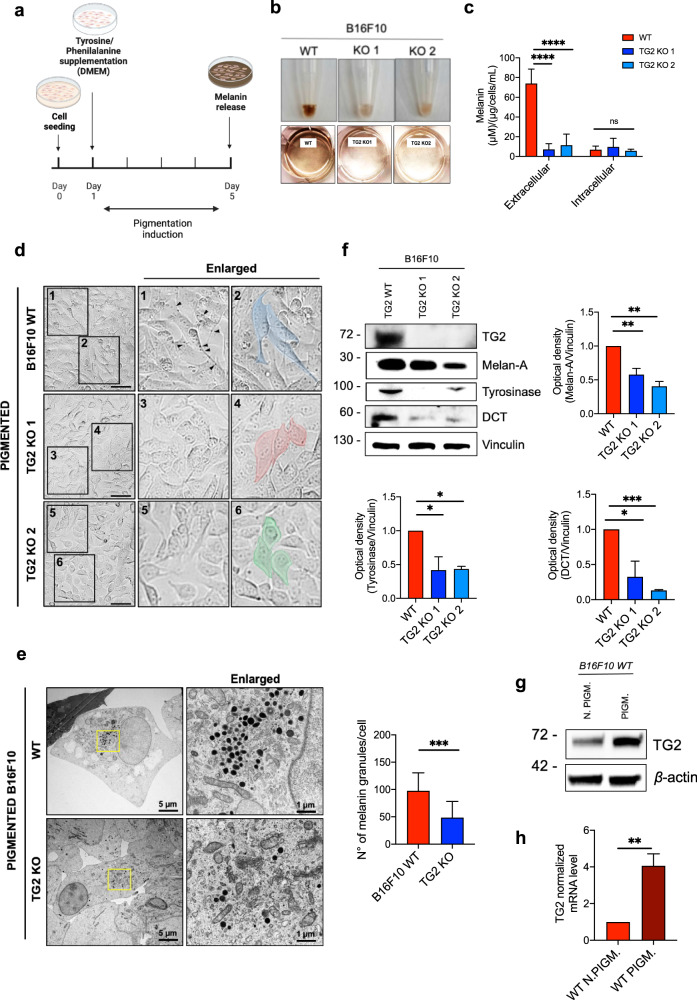
TG2 expression is required for melanogenesis in B16F10 cells. **a** Schematic representation of the employed pigmentation induction protocol, adapted from Sckoniecka et al., 2021 [46]. **b** Pictures of B16F10 WT and TG2 KOs cell pellets (upper panel) and media color (bottom panel) showing differential melanin (dark color) retainment and secretion between the samples. **c** Quantitative analyses of extracellular and intracellular melanin content, expressed in (µM)/(µg/cells/mL). Extracellular and intracellular melanin content was normalized on each well protein content. B16F10 WT was used as control during statistical analysis (number of independent biological replicates = 6). **d** Morphological analysis of B16F10 WT and TG2 KO clones following pigmentation induction by optical microscopy. Melanin granules are indicated with black arrows. Cellular shape is highlighted in blue, red, and green in WT, TG2 KO 1, and TG2 KO 2 respectively. Particularly, in B16F10 WT sample, cells acquire the typical differentiated dendritic shape with protrusions. Conversely, B16F10 TG2 KO cells maintain the typical melanoma spindle-like shape. Scale bar = 200 μm. **e** Transmission electron microscopy (TEM) images of ultrathin section of B16F10 WT and TG2 KO cells showing melanin-containing granules in the cytosol with relative granules per cell quantification. A higher magnification is reported in the right part of the panel (scale bars indicated in the pictures). Melanin granules (black) are enriched in the perinuclear area of the WT cell line. Isolated and dispersed fewer granules are visible in the KO condition. **f** Immunoblot analyses and relative densitometry of melanogenesis-related targets (Melan-A, Tyrosinase, and DCT) in B16F10 WT, TG2 KO 1, and TG2 KO 2 cells. Vinculin was used as loading control (number of independent biological replicates = 5). Immunoblot analysis (**g**) and relative mRNA levels quantified by qRT-PCR analysis (**h**) of TG2 expression in WT samples, following (PIGM.) or not (N. PIGM.) pigmentation. *β*-actin was used as a loading control in both immunoblot (number of independent biological replicates = 3) and qRT-PCR (number of independent biological replicates = 5). Statistical analyses of three or more groups were performed with One-Way ANOVA. Two-way ANOVA with Bonferroni’s test was used to compare the data with two variables. Statistical significance is specified with asterisks (^*^p < 0.05, ^**^p < 0.01, ^***^p < 0.001, ^****^p < 0.0001). Data are represented as mean ± SEM.

The hallmark of differentiated melanocytes and melanoma cells, which are derived from the neural crest, is the presence of melanin pigment, which strongly impacts the mechanical abilities of melanoma cells to spread [45]. Given the omics results, we hypothesized that TG2 could play a role in melanoma differentiation. To validate our hypothesis of a possible implication of TG2 in the process of differentiation, we decided to verify the pigmenting capacity of TG2 KO clones with respect to the WT cells, adapting the protocol by Skoniecka et al., 2021 [46]. To induce melanogenesis, cells were cultured in a melanin-precursors enriched medium (DMEM: L-Tyr = 72 mg/l, Phe = 66 mg/l) instead of the normal growth medium of B16F10 (MEM: L-Tyr = 52 mg/ml, Phe = 32 mg/ml) (Fig. 3a). Also, DMEM without phenol red was used for melanin quantification to prevent any interference with melanin absorbance measurements [47]. After subjecting the cells to the induction of pigmentation, we observed that only the WT cells can synthesize melanin, which gives a brown color to the pellet, whereas the pellets of the two KO clones remain white (Fig. 3b). Also, only WT medium acquires the typical brown color due to melanin release (Fig. 3b). The quantification of the extracellular melanin content demonstrates that the KO clones exhibit a severe alteration in the ability to secrete pigment. However, no significant alterations in intracellular melanin levels were observed (Fig. 3c). The typical dendritic morphology and dark pigmentation that distinguishes the differentiated state of melanoma cells were observed in pigmenting WT condition only (Fig. 3d). Also, the number of melanin granules quantified at the electron microscope is reduced by approximately 50% in the TG2 KO condition (Fig. 3e).

Melanin is formed through the activity of several melanogenesis-related proteins such as tyrosinase (Tyr) and dopachrome tautomerase (DCT, Trp-2) [48]. In this regard, the level of expression of Tyr and Dct was found strongly and significantly reduced in the TG2 KO clones both at the protein and transcript level (Fig. 3f, Fig. S4a). Melan-A, also known as MART 1 (Melanoma antigen recognized by T cells-Cloned gene) is a protein found both in the melanosomes and in the endoplasmic reticulum, which aids in the processing and transportation of PMEL (pre-melanosome protein), a key factor in the creation of melanosomes [49]. Here, we found a reduction of approximately 40% for the TG2 KO 1 clone and 60% for the TG2 KO 2 clone of Melan-A at the protein level (Fig. 3f).

**Fig. 4:**
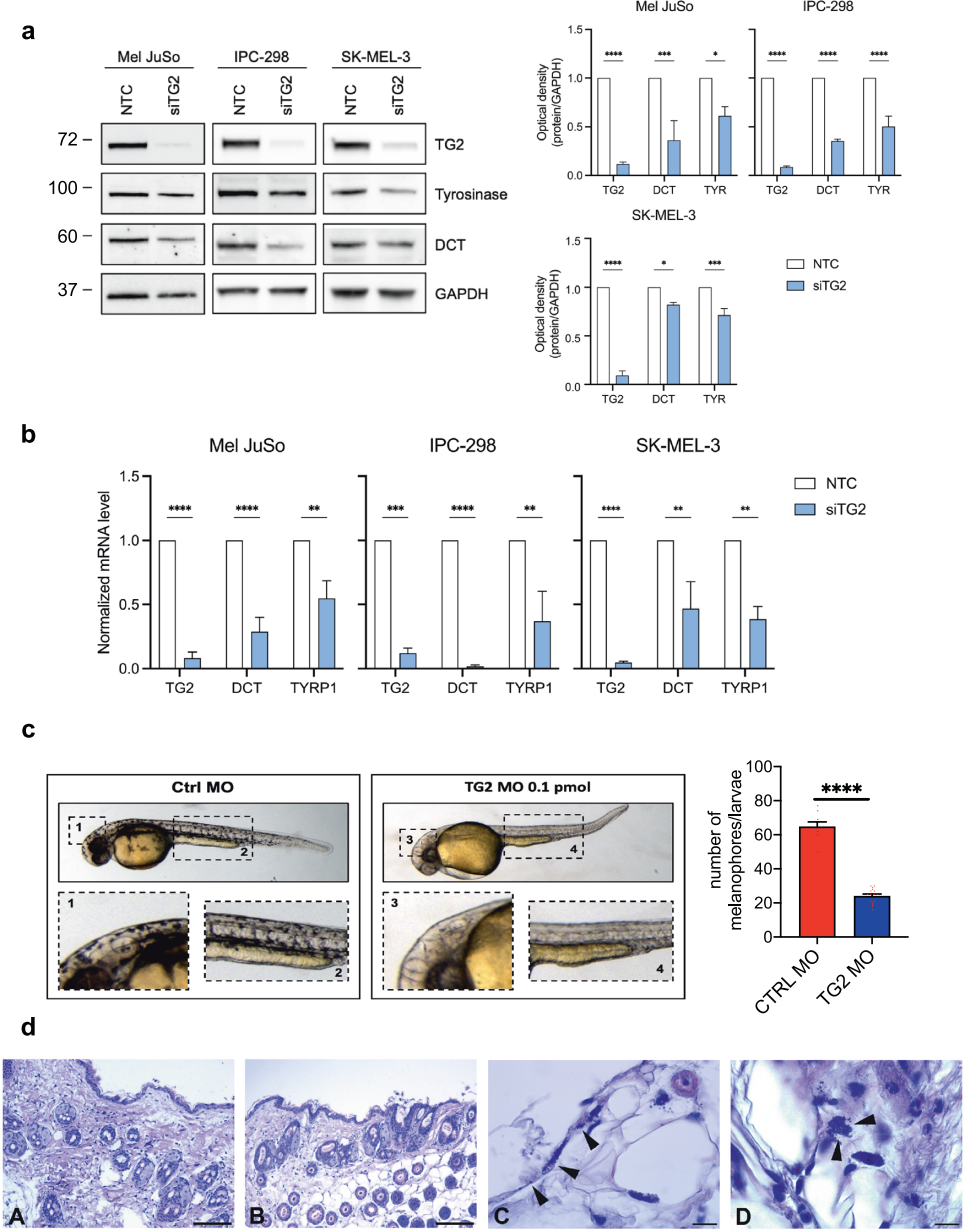
TG2 expression is required for pigmentation in human melanoma cell lines, and in vivo zebrafish and mouse models. **a** Immunoblot analyses and relative densitometry of melanogenesis-related targets (TG2, Tyrosinase, and DCT) in human melanoma cell lines Mel JuSo, IPC-298 and SK-MEL-3. GAPDH was used as loading control (number of independent biological replicates = 3). Statistical significance is specified with asterisks (^*^p < 0.05, ^***^p < 0.001, ^****^p < 0.0001). Data are represented as mean ± SEM. **b** Relative mRNA levels quantified by qRT-PCR analysis of TG2, DCT and TYRP1 expression in human melanoma cell lines Mel JuSo, IPC-298 and SK-MEL-3. *β*-actin was used as house-keeping gene in qRT-PCR (number of independent biological replicates = 3). Statistical significance is specified with asterisks (^*^p < 0.05, ^**^p < 0.01, ^***^p < 0.001, ^****^p < 0.0001). Data are represented as mean ± SEM. **c** Photos of zebrafish morphology at 48 hpf comparing melanophores formations in TG2 KD and Ctrl morphants and relative quantification. The zebrafish larvae were injected with 0.1 pmol of zTg2b antisense morpholino/embryo. Ctrl Morpholino (CtrlMO) was used as reference. Images were acquired with the same exposure, at 3.2X magnification. Statistical significance is specified with asterisks (^****^p < 0.0001). Data are represented as mean ± SEM. **d** Histology of mice skin: representative light micrographs of paraffin sections from C57BL/6 WT (A) and KO (B) mice skin, stained with hematoxylin and eosin, where the surface cornified layer and the numerous hair follicle are clearly identifiable; no detectable abnormalities are present in KO (B). C, and D depict magnifications. Melanin granules are visible along the dendritic extensions of melanocytes (arrowheads) are found in WT skin (shown in C). In TG2 KO skin melanin granules are mostly found in the perinuclear region of melanocytes (arrowheads) (shown in D). Scale bar: A, B = 150 µm; C, D = 12.8 µm.

Intriguingly, TG2 levels in the B16F10 WT cells significantly increase by a 3.5-fold change at protein level (Fig. 3g and S5c) and a 4-fold change at mRNA level (Fig. 3h) following pigmentation induction. Importantly, the pigmentation markers Tyrosinase and DCT were reduced, both at protein (Fig. 4a) and mRNA level (Fig. 4b), in human melanoma cell lines Mel JuSo, IPC-298 and SK-MEL-3 following downregulation of TG2 by siRNA. Altogether these results demonstrate that TG2 is induced when the pigmentation cascade is triggered in melanoma.

In addition to corroborate our findings, we checked the impact of TG2 expression on melanogenesis in two in vivo models. At first, we evaluated the development of melanophores in morphants *Danio rerio* embryos, namely *zTg2b*. The injection of 0.1 pmol of antisense TG2 morpholino in 48 hpf zebrafish embryos resulted in an evident defect in pigmentation, similar to an albino-like phenotype, as well as a significant reduction in the number of melanophores per larvae (Fig. 4c). Also, we performed a histological analysis to compare the epidermis of C57BL/6 WT mice to the one of TG2 KOs. Under physiological conditions, interfollicular melanin-producing melanocytes display melanin granules distributed along the dendritic structures of the cells, as indicated by the black arrows (Fig. 4d– panel C). On the contrary, in the skin from TG2 KO mice, in which were not observed morphological alteration compared to WT mice (Fig. 4d – panel A-B), melanin granules were mostly restricted to the cell body, in the perinuclear area (Fig. 4d – panel D), suggesting an impairment in melanin secretion.

Taken together, these data support our hypothesis that Transglutaminase type 2 expression is involved and required in the process of melanogenesis and that such mechanism is also conserved in vivo.

### TG2 interacts with MITF enabling its nuclear translocation

Considering the significant downregulation of the melanogenesis-related genes and the consequent albino-like phenotype of TG2 KO mutants in vitro and in vivo, we hypothesized that TG2 could play a role in the regulation of the MITF transcription factor. MITF is the master regulator of cell differentiation in melanocytes and melanoma. The activation of more than 100 genes required to regulate changes in cellular programs depend on MITF transcriptional activity [12]. In turn, MITF is regulated by numerous cellular pathways (Fig. S6a) on whose modulation the process of melanogenesis ultimately depends on. Thus, we decided to investigate the molecular bases behind the observed phenotype induced by loss of TG2 by evaluating the impact of TG2 expression on the MITF-activating pathways.

The α-melanocyte-stimulating hormone (α-MSH) is an endogenous peptide hormone of the melanocortin family that binds to the melanocortin-1 receptor (MC1R) on melanocytes to activate the transcription of MITF gene via PKA signaling cascade [50]. In 2014, Kim and colleagues reported that TG2 is required for the α-MSH mediated activation of melanin biosynthesis in human melanoma [51]. By contrast, we observed that upon stimulation with α-MSH also the TG2 KO clones are able to correctly synthesize and secrete melanin, as well as the WT line (Fig. S6b). Moreover, the usual dendritic shape of differentiated melanocytes is visible in the KO condition (Fig. S6b). Thus, we excluded that TG2 expression may have an impact on melanogenesis via the α-MSH cascade.

Furthermore, we did not detect significant alterations in any of the other known pathways that lead to the activation of MITF. In fact, neither the MAPK ERK1/2-p38 nor the canonical Wnt signaling cascade seem to be affected by loss of TG2 (Fig. S6c). Also, treatment with the CHIR 99021 activator of the canonical pathway of Wnt signaling can induce the pigmentation in TG2 KO clones (Fig. S6d), corroborating the idea that this pathway is also not affected by loss of TG2.

**Fig. 5:**
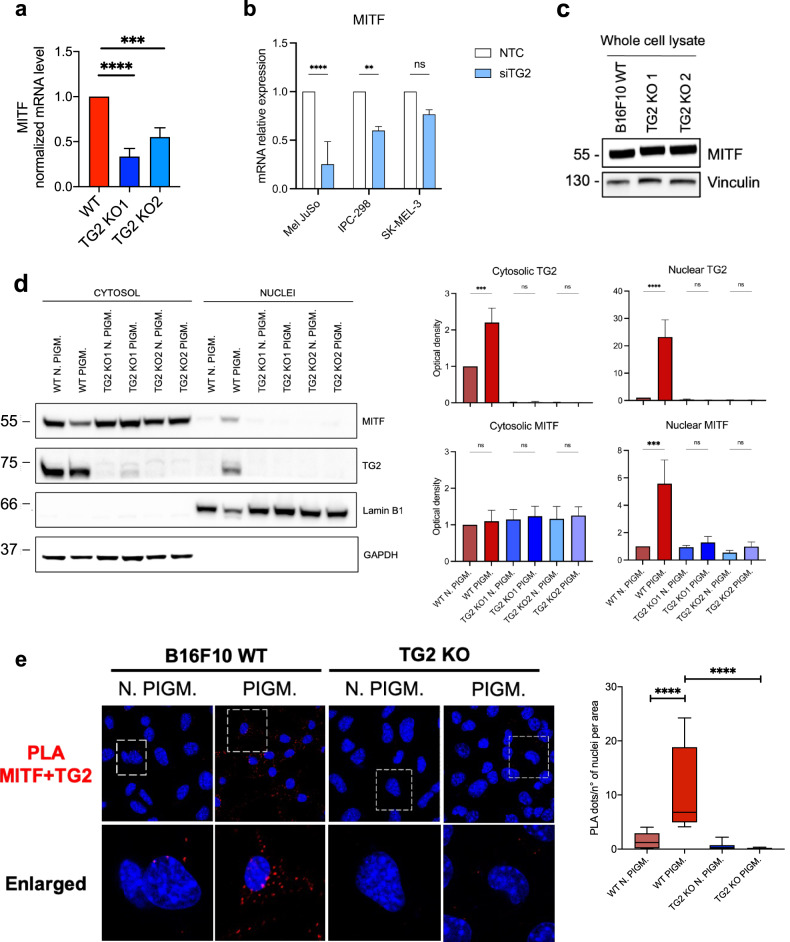
TG2-MITF interaction is required for MITF nuclear translocation. Relative mRNA levels quantified by qRT-PCR analysis of MITF expression in B16F10 WT, TG2 KO 1 and TG2 KO 2 (**a**) and in human melanoma cell lines Mel JuSo, IPC-298 and SK-MEL-3 (**b**). *β*-actin was used as house-keeping gene in qRT-PCR (number of independent biological replicates = 3–5). **c** Immunoblot analysis of MITF in B16F10 WT, TG2 KO 1 and TG2 KO 2. Vinculin was used as loading control (number of independent biological replicates = 3). **d** Cytosolic-nuclear fractionation assay and relative densitometric analyses evaluating the expression and localization of MITF and TG2 in WT and KO clones, following (PIGM.) or not (N. PIGM.) pigmentation induction. Vinculin and Lamin C were used as loading controls, respectively marking the cytosolic and the nuclear fractions (number of independent biological replicates = 3). **e** In situ Proximity Ligation Assay (PLA) showing the interaction between MITF and TG2 in B16F10 WT and TG2 KO conditions, following or not pigmentation induction. Each red spot represents a single interaction. DNA was stained with DAPI (in blue). Quantification of dots per cells is represented in the graph on the right. Statistical analyses were performed with One-Way ANOVA and specified with asterisks (^**^p < 0.01, ^***^p < 0.001, ^****^p < 0.0001). Data are represented as mean ± SEM.

After excluding the involvement of TG2 in the canonical mechanisms that lead to melanogenesis, we hypothesized a direct regulation of the MITF transcription factor by TG2. Consistent with the downregulation of melanogenesis-related genes, ablation of TG2 leads to a reduction of MITF mRNA levels, both in B16F10 melanoma TG2 KO clones (Fig. 5a), as well as in human melanoma cell lines Mel JuSo, IPC-298 and SK-MEL-3 following downregulation of TG2 by siRNA (Fig. 5b). However, we did not detect significant alterations in MITF protein levels (Fig. 5c). This result could be explained by the fact that being a transcription factor, MITF has a high turnover within melanocytes. Having found no significant differences in MITF protein levels, we wondered if there was an alteration in its transcriptional activation. To address this issue, we first performed a cell fractionation assay to study MITF subcellular localization and check whether an impairment of its nuclear translocation may have occurred. By cyto-nuclear fractionation, we observed that MITF significantly accumulates in the nuclear fraction of B16F10 WT pigmenting cells (Fig. 5d). This is in line with what is expected, since during pigmentation MITF becomes transcriptionally active to induce the synthesis of the melanogenesis-related genes. Intriguingly, MITF does not accumulate at the nuclear level following the induction of pigmentation in the TG2 KO cells, suggesting that loss of TG2 may impair its nuclear translocation and activation. This data could justify the downregulation of the expression of the melanogenesis genes and the subsequent loss of pigmentation in both TG2 KO cells (Fig. 5d).

Interestingly, we noticed that upon induction of pigmentation, TG2 is also found in B16F10 WT nuclei (Fig. 5d). In this regard, we recently demonstrated that TG2 can play a scaffold role by binding to β-catenin and allowing its transport to the nucleus, contributing to the regulation of the Wnt signaling [52]. Given the colocalization between TG2 and MITF in the pigmenting B16F10 WT nuclear fraction, we hypothesized that TG2 could also play a shuttle function to regulate MITF nuclear transport and subsequent melanogenesis related genes activation. To check this hypothesis, we addressed the presence of a direct interaction between TG2 and MITF both by Proximity Ligation Assay (PLA) and Co-IP. Consistent with our hypothesis, PLA analyses demonstrated that a co-localization between TG2 and MITF occurs in B16F10 WT cells and significantly increases during melanogenesis (Fig. 5e). This result was further confirmed by the Co-IP assay between TG2 and MITF on the nuclear enrichment of the pigmented B16F10 WT cells (Fig. S7).

Overall, these data suggest that TG2 ablation could disrupt the correct nuclear translocation of MITF during pigmentation, which in turn leads to an impairment in melanin production of the TG2 KO clones.

### TG2 ablation leads to increased invasiveness both in vitro and in vivo

Beside its role as master regulator of pigmentation, MITF is central to the control of melanoma plasticity and heterogeneity, named “phenotype switching”, introduced by Hoek et al. in 2008 [53]. According to the phenotypic switching model, there exist two main programs in which melanoma can interconvert, namely the differentiated/proliferative and the undifferentiated/invasive [54].

Given the loss of pigmentation (Figs. 3–4) and low levels of nuclear MITF (Fig. 5), as well as the increase in metastatic and invasive markers that characterize the TG2 KO clones (Fig. 2g, l), we speculated that TG2 could play a role in regulating the transition between melanoma plasticity signatures. For instance, TG2 KO clones display a significant downregulation of *Cited1* mRNA levels, a marker of the proliferative state (Fig. S4b). Loss of Cited1 is correlated with reduced MITF expression and worse prognosis for patients with primary SKCM [13]. Also AXL, a member of the TAM tyrosine kinase receptor family, plays a central role in the mesenchymal motif by regulating cell proliferation, EMT, migration, and immune responses in melanoma cells. The expression of AXL is inversely related to that of MITF, so that MITF^high^/AXL^low^ (proliferative) and MITF^low^/AXL^high^ (invasive) populations contribute mostly to intratumor heterogeneity in melanoma and, thereby, resistance to therapy [55]. In line with this, we found significant upregulation of AXL mRNA levels in both TG2 KO clones (Fig. S3i). In addition, TG2 KO clones display a significant increase of the EMT marker FN1, both at the mRNA (Fig. S4c) and protein (Fig. S3j) levels.

**Fig. 6:**
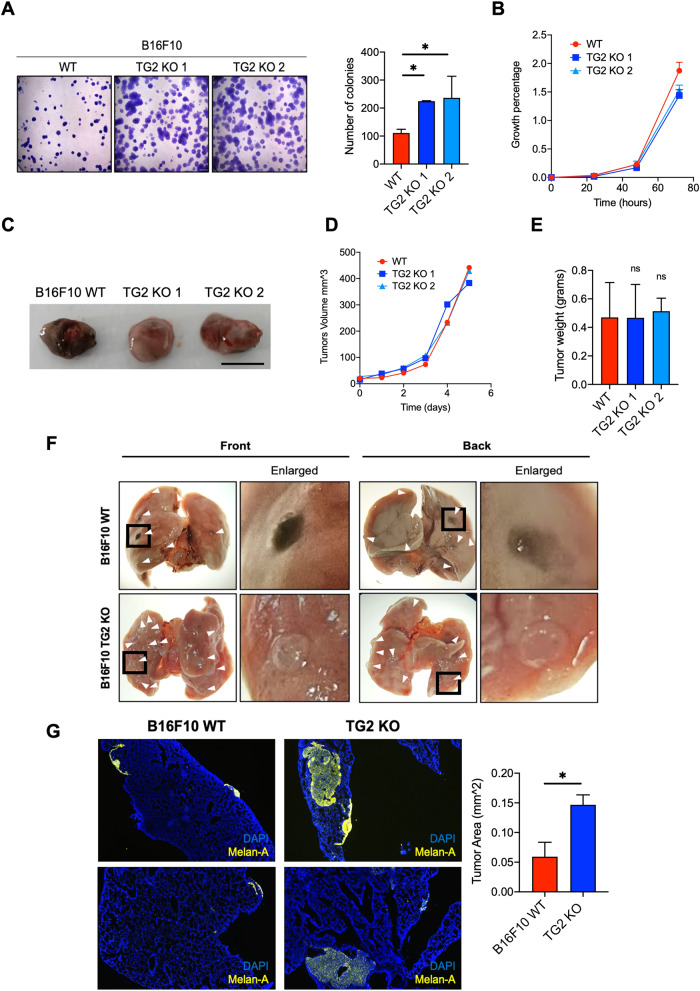
Characterization of TG2 KO melanoma tumorigenic potential in vitro and in vivo primary tumors and metastatic formations. **A** Colony formation assay with quantification of the number of colonies per sample (number of independent biological replicates = 5). The number of colonies was assessed with ImageJ (One-Way ANOVA, ^*^p < 0.05). **B** Growth curve comparing the proliferation rate (expressed in growth percentage/hours) between B16F10 WT and the two TG2 KO clones. Parental B16F10 and Cas9-transfected cells displayed the same proliferation rate (not shown, number of independent biological replicates = 3). **C**–**E** Analysis of in vivo primary tumor growth from C57BL/6 WT orthotopic mice models after injection with the indicated cell lines. 4 animal models were injected for each group. Excised tumors are reported (**C**), showing a difference in pigmentation relatable to the different melanin content between TG2 WT and KO clones. Tumors were measured daily to assess the growth volume (**D**) and were weighted after the excision (**E**). **F** Analysis of lung experimental metastasis formation in C57BL/6 mice induced from B16F10 WT and TG2 KO tail vein injection. A picture of the front and back of mice lungs is reported for each condition. White arrows point to the experimental metastatic processes. **G** Multiplex IHC on lung experimental metastasis tissue of C57BL/6 mice induced from B16F10 WT and TG2 KO tail vein injection and relative tumor area quantification. Melanoma cells infiltration in tissues was visualized by anti-Melan-A staining (in yellow). DNA was stained with DAPI (in blue). Multiple 4 µm sections from 4 mice per conditions were used for the statistical analyses. Statistical analyses of three or more groups were performed with One-Way ANOVA. Two-way ANOVA with Bonferroni’s test was used to compare the data with two variables. Statistical significance is specified with asterisks (^*^p < 0.05). Data are represented as mean ± SEM.

Given these premises, we hypothesized that TG2 expression could prevent the onset of the mesenchymal state of melanoma cells. Thus, we proceeded with the evaluation of the impact of TG2 expression on tumor growth in vitro and in vivo. As reported in Fig. 6A, in vitro analysis of the clonogenic potential showed a significant increase in the number of TG2 KO colonies (about double those formed starting from WT). By contrast, no significant changes were found in proliferation levels between the three cell lines (Fig. 6B). The discrepancy between these two results can be explained by the very definition of “proliferation assay” and “colony formation assay”, two techniques that highlight different aspects of tumor growth. Indeed, the colony formation assay is based on the ability of a single cell to grow into a colony, undergoing unlimited divisions [56]. Conversely, the proliferation rate concerns the capability of the entire population to increase its number, by continuously doubling. Furthermore, we found no differences in the growth, volume, and weight of primary tumors injected in C57BL/6 WT mice (Fig. 6C–E). Noticeably, also the primary tumors deriving from TG2 KO 1 and TG2 KO 2 clones are de-pigmented compared to the counterpart deriving from the B16F10 WT cells (Fig. 6C).

To measure the invasive capacity of the clones in vivo we injected tumor cells into the tail vein of the mice, and we followed the formation of lung experimental metastasis. In line with our hypothesis, individual KO experimental metastasis appear larger and paler than that of the WT ones (Fig. 6F). Given the difficulty in quantifying white metastases from KO clones, we decided to take advantage from immunohistochemical staining to precisely quantify the metastatic area covering the lungs. According with our hypothesis, immunofluorescence analysis with specific labeling for melanoma cells (anti-Melan-A staining) revealed that the area covered by TG2 KO experimental metastasis in the lungs are larger than those formed after injection of WT cells (Fig. 6G).

Overall, these data suggest that TG2 expression has a positive role in preventing and regulating melanoma invasive capability, by supporting the differentiated/proliferative state and consequently reducing metastasis formation.

## Discussion

Melanoma progression, metastasis formation, and therapy resistance are related to the capacity of cells to switch from a differentiated towards a dedifferentiated/invasive phenotype. In many cancers, the acquisition of invasive hallmarks is a process called “epithelial to mesenchymal transition” (EMT). In melanoma, the plasticity between proliferative and invasive states is defined as “phenotype-switching”. The first, which has also been described as “differentiated”, “epithelial-like”, and often “therapy-sensitive”, is characterized by a hyper-activation of MITF (MITF^high^) that supports a strong degree of cellular differentiation and proliferation. Conversely, the invasive phenotype defined as “undifferentiated/dedifferentiated”, “mesenchymal-like”, and often “therapy-resistant”, is characterized by low levels of MITF (MITF^low^) which lead to an increase in metastatic potential at the expense of cell proliferation [1, 7, 8, 10].

The regulation of the phenotypic plasticity of melanoma is very complex: only in recent years cancer research has attempted to better define its functioning, which has led to the identification of numerous factors that take part in such process. For instance, the antithetical expression between receptor tyrosine kinase AXL and MITF has led to the definition of the proliferative phenotype as MITF^high^/AXL^low^, and the invasive one as MITF^low^/AXL^high^ [55]. Another relevant modulator of SKCM plasticity is represented by Cited1, a non-DNA binding transcriptional co-regulator whose expression can distinguish the “proliferative” from “invasive” signature, so that loss of Cited1 is correlated with reduced MITF expression and with a worse outcome [13]. However, we are still far from understanding all the factors that take part in the regulation of this intricate process.

In this study, we identified a new player in the management of melanoma heterogeneity, namely Transglutaminase type-2 (TG2). TG2 is a multifunctional enzyme, whose role in cancer is controversial and tissue dependent [20]. Still, the part played by TG2 in EMT processes has already been highlighted in several contexts [20]. To shed light on the controversies regarding its role in cancer disease, we performed bioinformatics analyses on public cancer datasets which unveiled that the prognostic role of TG2 is generally negative, except for SKCM, the only tumor type in which TG2 expression is associated with a positive prognosis, as we already mentioned in Muccioli et al., 2022 [21]. In the same article, we reported that TG2 expression was upregulated in metastatic samples compared to primary tumors. This apparent discrepancy between the level of TG2 expression in primary and metastatic melanoma and its prognostic role in patient survival can be explained by taking two key elements into consideration: I) the melanoma RNAseq data contained in the TCGA database refer to bulk tumors, in which the expression of genes by not only tumor cells but also all stromal components is taken into account, including endothelial cells, fibroblasts, CAFs, etc. This means that the level of expression of a gene can be influenced not only by the tumor but also by the cumulative effect of all the components of the tumor nest [57]. II) In Muccioli et al., 2022 we speculated that the beneficial role of TG2 expression in melanoma is also due to an effect of recruitment and activation of the immune system which, in this way, could favor a positive response to therapeutic treatment [58].

In the present work we tried to deepen the role of TG2 in SKCM. First, we ablated its expression in a commonly used murine melanoma model. Our analyses performed on the generated model unveiled TG2 implications in the regulation of two processes determining the phenotypic status of melanoma. On the one hand, we showed that TG2 is required for pigmentation and that its function is conserved in animal models. In 2014 Kim and colleagues suggested TG2 involvement in the regulation of melanogenesis in human melanoma cells, without delving into the molecular mechanism [51]. According to the phenotype switching model [53], pigmentation is a hallmark discriminating melanoma differentiated state, as melanoma cells acquiring the invasive signature decrease the expression of melanogenesis markers, which strongly impact on the mechanical abilities of melanoma cell to spread [45]. Also, being melanoma the only type of tumor capable of pigmenting, this result could partially give insights into the positive role of TG2 exerted in SKCM tumor only.

On the other hand, we demonstrated that loss of TG2 expression leads to an increase in the invasive capacity and extracellular environment remodeling of the tumor that characterize melanoma invasive state [53]. These findings correlate with the increase in the number of lung metastases found in TG2 KO melanoma-injected mice compared to the WT line. In addition, loss of Cited1 expression accompanied with an increase in the EMT hallmark Fibronectin 1 and the antithetic expression between AXL and MITF that we found in the TG2 KO model, collectively support the idea of a possible participation of TG2 in the modulation of melanoma heterogeneity via the phenotype switching.

To shed light on the molecular mechanisms behind our findings, we focused our attention on MITF. Indeed, both melanogenesis and invasive capabilities depend on MITF levels and activation in melanoma [53].

**Fig. 7:**
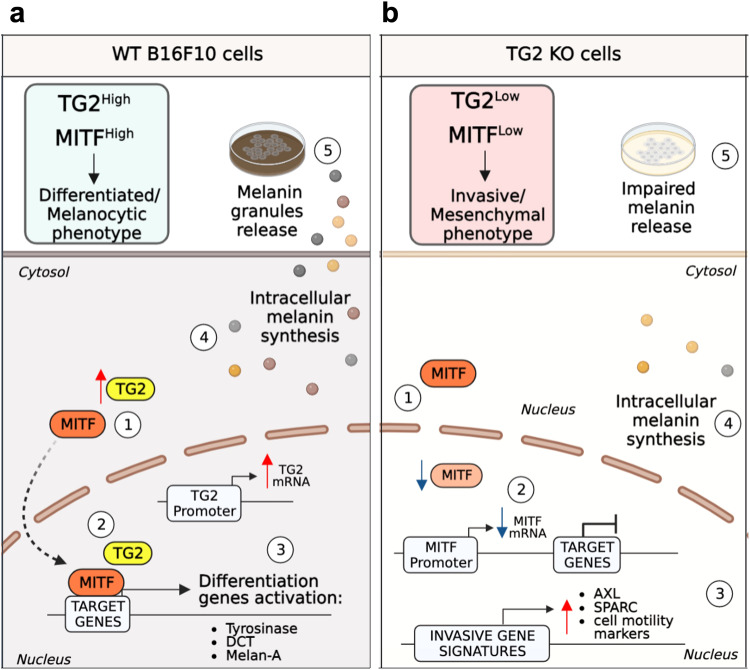
Schematic representation of the hypothesized working model. **a** TG2 expression increases during pigmentation of melanoma cells. Also, following pigmentation, TG2 interaction with MITF allows the transcription factor nuclear translocation and its subsequent transcriptional activation by synthesis of the melanogenesis-related genes (Tyr, Dct, Melan-A, etc), which allow intracellular melanin synthesis and extracellular secretion. Thanks to TG2, MITF^High^ levels enable the maintenance of the melanocytic/differentiated state. **b** Loss of TG2 inhibits correct MITF nuclear translocation, contributing to a downregulation of melanogenesis and melanoma de-differentiation. Loss of TG2 increases MITF^Low^/AXL^High^ ratio, switching melanoma cells to the mesenchymal/invasive phenotype, increasing its metastatic capacity by promoting cell motility, alteration of cell-adhesion molecules, and extracellular remodeling. Schematic representation was created with BioRender.com.

Particularly, we observed that during pigmentation TG2 translocates into the nucleus of melanoma cells following an increase in its expression levels. Furthermore, we demonstrated that during this process, TG2 binds MITF to facilitate its nuclear translocation, acting as a protein scaffold and indirectly favoring its transcriptional activity (Fig. 7). This shuttle / scaffold function of TG2 between cytosol and nucleus has already been observed previously for other proteins that need to reach the nucleus to exploit their activity [59]. For instance, we recently demonstrated that TG2 can support nuclear localization of the β-catenin, a key player of the canonical Wnt signaling pathway. As observed with MITF, TG2 can physically interact with the β-catenin [52]. Although one of the best-studied pathways involved in melanoma cell plasticity is represented by canonical Wnt signaling-dependent up-regulation of MITF expression [60], this effect has not been observed in our experiments, since β-catenin expression was not depleted in TG2 KO clones (Fig. S6c). In addition, increased nuclear TG2 can bind to HIF1β so decreasing the hypoxia-responsive element-dependent upregulation of pro-apoptotic proteins, thereby protecting neuronal cells from hypoxia-induced death in ischemia and stroke [61, 62]. Moreover, TG2 can associate and mediate the nuclear translocation of the p65 subunit of NFkB [63], the receptor VEGFR-2 [64], and HSF-1 [65].

Even though nuclear TG2 comprises only a small proportion (around the 5–7%) of the total cellular amount, it has attracted increasing interest because of its great importance in modulating various cellular processes. Indeed, being associated with the euchromatin [66], even minor changes in nuclear TG2 levels and/or its activities may result in significant effects on gene regulation, and thereby cellular responses, during the pathogenesis of diseases and their treatment.

Given the growing understanding of the regulatory networks that drive phenotype switching and its role in melanoma metastasis and therapy resistance, one strategy is to block or reverse the invasive switch by directing melanoma cells towards the more therapy-sensitive proliferative/melanocytic state [67]. Considering our work, tuning of cytosol to nuclear TG2 translocation could offer new perspectives to address melanoma vulnerabilities. Although more studies are needed to better evaluate the potential of TG2 in SKCM, we believe that our work may pave the way for identifying novel winning strategies to target melanoma phenotype switching and sensitize this tumor to treatments.

## Methods

### Ethics approval and consent to participate

Zebrafish (animals and embryos) were maintained according to standard rules and procedures (https://zfin.org). All animal manipulation procedures were conducted according to the Local Ethical Committee at the University of Padua and National Agency (Italian Ministry of Health Authorization number 407/2015-PR), and with the supervision of the Central Veterinary Service of the University of Padova (in compliance with Italian Law DL 116/92 and further modifications, embodying UE directive 86/609).

Mice experiments were carried out according to the Local Ethics Committee of the University of Padua and the National Agency, and under the supervision of the Central Veterinary Service of the University of Padova (in compliance with Italian law DL 116/92 and further modifications, embodying UE directive 86/609), authorization n. 144/2022-PR. Male wild-type mice (8 weeks old) in the C57BL6/J background were kept on a 12 h light/dark cycle at controlled temperature and humidity, with standard food (4RF21, Mucedola Srl, Italy) and water provided ad libitum and environmental enrichments.

### Cell cultures

In vitro experiments were performed with murine melanoma B16F10 cells and human melanoma cell lines Mel JuSo, IPC-298 and SK-MEL-3. B16F10 cells were cultured in Minimum Essential Medium (MEM, Invitrogen). Mel JuSo, cells were cultured in RPMI (Invitrogen). IPC-298 and SK-MEL-3 cells were cultured in Dulbecco’s Modified Eagle Medium (DMEM, Invitrogen). All media were supplemented with 10% fetal bovine serum (FBS, BioSpa S.p.A.), 100 U/ml penicillin and 100 U/ml streptomycin (Life Technologies), 1X non-essential amino acids (LifeTechnologies). For the pigmentation experiments we used the white Dulbecco’s modified Eagle medium (DMEM, Invitrogen) supplemented with 10% fetal bovine serum (FBS, BioSpa S.p.A.), 10 mM HEPES (Life Technologies), 100 U/ml penicillin and 100 U/ml streptomycin (Life Technologies), 1X non-essential amino acids (Life Technologies). All cells were maintained at 37 °C and 5% CO2.

### Kaplan–Meier curves and Hazar Ratio assessment

Kaplan–Meier curves were retrieved using GEPIA (gene expression profiling interactive analysis) (http://gepia.cancerpku.cn/). GEPIA is an interactive web application for gene expression analysis based on 9736 tumors and 8587 normal samples [22]. GEPIA was used to analyze the expression of TG2 in 32 different histotypes of tumors and its effects on survival rate by means of the Kaplan–Meier analysis tool. We divided samples between high and low TG2 expression groups according to the quartile TGs mRNA levels to analyze overall survival (Log2FC cutoff: 1; *p*-value cutoff: 0.01, group cutoff selected median, cutoff-high (%): 25; cutoff-low (%): 75).

To assess the Hazard Ratio (HR), Survival Genie software was employed (https://bbisr.shinyapps.winship.emory.edu/SurvivalGenie/). Survival Genie is an open source that contains 53 datasets of 27 distinct malignancies from 11 different cancer programs related to adult and pediatric cancers [27]. The tool provides results with univariate Cox proportional hazards model. To assess HR based on TG2 expression in LGG, GBM, KIRC, LUSC, and SKCM primary tumors, we used the Cutp option for the Cut-off establishment (the cut-point is estimated based on martingale residuals using the *survMisc* package to stratify patients into high and low groups).

### Generation of TG2 KO B16F10 clones using the CRISPR/Cas9 technology

The CRISPR/Cas9 approach followed the guidelines described by Ran and colleagues [28] using the pSpCas9n (BB) −2A-Puro (PX459) (Addgene Plasmid #48139 Zhang Lab). The sgRNA probes were designed to excise the portion of the *TGM2* gene that includes the transcription initiation site (TSS), the translation initiation site, and all of exon 1. Particularly, the sgRNAs used are listed in Table 1.

Single guides were ligated into the PX459 vector at the BbsI (NEB) restriction site under the U6 promoter and verified by sequencing. Different couples of single guides were used to transfect cells. sgRNAs 1 and 2 map upstream the TSS, while sgRNA 3 maps downstream *TGM2* exon 1. Pair 1 + 3 was used to generate TG2 KO 2 clone and creates a deletion of approximately 264 base pairs, while pair 2 + 3 was used to generate TG2 KO 1 clone and creates a deletion of 397 bps.

For B16 F10 wild-type cell line transfection, the TransIT®-LT1 transfection reagent (Mirus) was used. Briefly, cells were seeded in a 6-well plate. After 24 h, cells were transfected with 1.25 µg of plasmid DNA containing the gRNAs. Puromycin 2.5 µg/ml (Gibco) was added to the cell culture medium 48 h after transfection and kept for 96 h. After selection, cells were serially diluted to obtain single-cell clones. Clones were expanded and cells were collected and centrifuged for 5 min at 600 *g*. After removing the media, DNA was extracted from the pellet using the MyTaq Extract-PCR kit (Meriadin Bioscience) following manufacturer’s instructions. A control PCR was performed with primers reported in Table 2.

To test TG2 expression in WT vs KO clones, qRT-PCR analyses was employed. Briefly, 500,000 cells were seeded in 6-well plates. One day after seeding, total RNA was extracted using the Qiagen RNeasy Mini Kit (Qiagen, #74104). Genomic DNA was digested following the manufacturer’s instructions. 2 µg of RNA were retrotranscribed using the SuperScript IV Reverse Transcriptase (Thermofisher, #18091050) following manufacturer’s instructions. cDNA was diluted 1:20 and amplified in qRT-PCR using SYBR Green PCR Master Mix (Thermofisher, #4309155). Actin was used as housekeeping control. CT values were first normalized with respect to the housekeeping genes (ΔCT) and next compared to the control sample (ΔΔCT). The relative normalized expression is indicated in the figures. Primers listed in Table 3 were used for qRT-PCR amplification.

As further validation, B16F10 KO clones were also tested through Western Blot analyses. Vinculin, *β*-actin, GAPDH or Latin A/C were used as housekeeping gene. TG2 protein level was normalized on the B16F10 WT cell line.

### Mass spectrometry

#### Sample preparation SP3 and TMT labeling, OASIS

For the Proteomics sample preparation, two technical replicates and three biological replicates for each sample condition (B16F10 WT, TG2 KO 1, and TG2 KO 2) were collected. Briefly, 5 × 10^5^ cells were seeded in a 6-well plate. The following day, cells reaching 90% confluence were washed and proteins extracted using the LUC lysis buffer (25 mM Tris, 2.5 mM EDTA, 10% glycerol, 1% NP-40) supplemented with DTT and protease inhibitors. Samples were centrifuged 15 min at 15,000 *g*, and supernatant was subjected to Benzonase (Cat# 9025654, Sigma-Aldrich) treatment (30 min at 37 °C) to remove nuclei acids. The reduction of disulphide bridges in cysteine-containing proteins was performed with dithiothreitol (56 °C, 30 min, 10 mM in 50 mM HEPES, pH 8.5). Reduced cysteines were alkylated with 2-chloroacetamide (room temperature, in the dark, 30 min, 20 mM in 50 mM HEPES, pH 8.5). Samples were prepared using the SP3 protocol [68] and trypsin (sequencing grade, Promega) was added in an enzyme to protein ratio 1:50 for overnight digestion at 37 °C. The peptides were labelled with TMT11plex [69] Isobaric Label Reagent (ThermoFisher) according to the manufacturer’s instructions. Samples were combined for the TMT11plex and for further sample clean up an OASIS® HLB µElution Plate (Waters) was used. Off-line high pH reverse phase fractionation was carried out on an Agilent 1200 Infinity high-performance liquid chromatography system, equipped with a Gemini C18 column (3 μm, 110 Å, 100 × 1.0 mm, Phenomenex).

#### LC-MS/MS acquisition

An UltiMate 3000 RSLC nano LC system (Dionex) fitted with a trapping cartridge (µ-Precolumn C18 PepMap 100, 5 µm, 300 µm i.d. x 5 mm, 100 Å) and an analytical column (nanoEase™ M/Z HSS T3 column 75 µm x 250 mm C18, 1.8 µm, 100 Å, Waters) were used. The trapping was carried out with a constant flow of trapping solution (0.05% trifluoroacetic acid in water) at 30 µL/min onto the trapping column for 6 min. Subsequently, the peptides were eluted via analytical column running solvent A (0.1% formic acid in water, 3% DMSO) with a constant flow of 0.3 µL/min, with an increasing percentage of solvent B (0.1% formic acid in acetonitrile, 3% DMSO). The outlet of the analytical column was coupled directly to an Orbitrap Fusion™ Lumos™ Tribrid™ Mass Spectrometer (Thermo) using the Nanospray Flex™ ion source in positive ion mode.

The peptides were introduced into the Fusion Lumos via a Pico-Tip Emitter 360 µm OD x 20 µm ID; 10 µm tip (New Objective) and an applied spray voltage of 2.4 kV. The capillary temperature was set at 275 °C. The full mass scan was acquired with mass ranges of 375–1500 m/z in profile mode in the orbitrap with a resolution of 120,000. The filling time was set at a maximum of 50 ms with a limitation of 4 × 10^5 ^ions. Data-dependent acquisition (DDA) was performed with the resolution of the Orbitrap set to 30,000, with a fill time of 94 ms and a limitation of 1 × 10^5 ^ions. A normalized collision energy of 38 was applied. The MS2 data was acquired in profile mode.

#### MS data analysis – Isobarquant

IsobarQuant and Mascot (v2.2.07) were used to process the acquired data, which was searched against a Uniprot Mus Musculus proteome database (UP000000589) containing common contaminants and reversed sequences. The following modifications were included in the search parameters: Carbamidomethyl (C) and TMT11 (K) (fixed modification), Acetyl (Protein N-term), Oxidation (M) and TMT11 (N-term) (variable modifications). For the full scan (MS1), a mass error tolerance of 10 ppm was set and for MS/MS (MS2) spectra of 0.02 Da. Further parameters were established: trypsin as protease with an allowance of a maximum of two missed cleavages: a minimum peptide length of seven amino acids; at least two unique peptides were required for protein identification. The false discovery rate at the peptide and protein level was set to 0.01.

#### Mass spectrometry data analysis

The raw IsobarQuant output files (protein.txt – files) were processed using the R programming language (www.r-project.org). Only proteins that were quantified with at least two unique peptides and identified in all mass spec runs were considered for analysis. Raw reporter ion intensities (signal_sum columns) were first cleaned for batch effects using limma [70] and further normalized using vsn (variance stabilization normalization [71]. Missing values were imputed with the ‘knn’ method using the Msnbase package [72]. The differential expression of the proteins was tested using the limma package. The replicate information was added as a factor in the design matrix given as an argument for the limma lmFit function. Furthermore, the imputed values were given a weight of 0.05 in the ‘lmFit’ function. A protein was annotated as a hit with a false discovery rate (fdr) smaller than 5% and a fold change of at least 100% and as a candidate with a fdr below 20 % and a fold-change of at least 50%.

#### Bioinformatic analysis

The list of the selected proteins was used to identify significantly enriched functional categories. Enrichment analyses were performed using clusterprofiler R package [73] on Gene Ontology (GO) categories of biological processes (BP). GO of molecular function (MF) and cellular component (CC) were retrieved using the online software g Profiler [74], a web server for functional enrichment analysis and conversions of gene lists. False discovery rate (FDR) was used to control for multiple testing. A threshold of 0.01 (FDR < 0.01) was used to identify significantly enriched GO terms. Semantic similarity distance as implemented in rrvo R package *(*https://ssayols.github.io/rrvgo*)* was implemented to reduce redundancy of the significant GO terms. Bar plot, dot plot, heat maps, and tables were used to graphically summarize and report the results.

### RNA-seq analyses: sample preparation, alignment, pre-processing and differential gene expression

For the RNAseq sample preparation, two technical replicates and three biological replicates for each sample condition (B16F10 WT and TG2 KO 2) were collected. Briefly, 5 × 10^5^ cells were seeded in a 6-well plate. The following day, cells reaching 90% confluence were washed and total RNA was extracted with Qiagen RNeasy Mini Kit (Qiagen, #74104). Genomic DNA was digested following the manufacturer’s instructions.

Reads were aligned to the reference genome with STAR (v 2.7.10a) [75, 76] and quantified with RSEM (v1.3.1). The indexed genome was built with RSEM starting from Ensembl’s Mus Musculus DNA primary assembly (release 106). For all of the 9 aligned samples we obtained a percentage of uniquely mapped reads between 73.12% and 79.93%, while the numbers of uniquely mapped reads were between 24600173 and 74021101.

After the quantification, data were filtered keeping only genes with at least 20 counts in three different samples.

To identify the differentially expressed genes we used the edgeR R package [77]. We provided as input the filtered raw counts with the design matrix defined by the dichotomous variables for the different clones. The TMM normalization was applied to the samples. False Discovery Rate (FDR) less than 0.01 was used to select significantly differential genes (DEG).

DEG were used to perform enrichment analysis (separately for up and down-regulated genes) on Gene Ontology with clusterProfiler and ReactomePA R packages. For the enrichment analysis the universe was set as the list of the genes with at least one count in one sample. Adjusted *p-*values less than 0.1 was used to select significant gene sets and pathways.

Cluster analysis was performed using the pheatmap R package with the complete linkage method with euclidean distances.

Raw data have been deposited at SRA and are available at the accession number reported as follow: RNAseq raw data - SubmissionID: SUB12302131, BioProject ID: PRJNA904573

### Pigmentation inducing treatments on B16F10 cells

B16F10 are cultured in MEM (Minimum Essential Medium). To induce pigmentation, B16F10 cells were grown in DMEM (Dulbecco Modified Minimal Essential Medium), according to Skoniecka et al., 2019 [46]. Both media are supplemented with 10% FBS and antibiotics: penicillin (100 U/ml) and streptomycin (100 μg/ml). The cultures were maintained at 37 °C in 5% CO2. Both media are recommended for in vitro melanoma cells culture. DMEM medium contains more (72 mg/l) L-tyrosine, the basic amino acid for melanin synthesis, than MEM (52 mg/l). Media differ also in phenylalanine level (66 mg/l in DMEM, 32 mg/l in MEM), which could be hydroxylated into L-tyrosine in the presence of L-phenylalanine hydroxylase. DMEM, as a medium with higher L-tyrosine content, is indicated as a factor able to induce melanization in amelanotic melanoma cells [45, 78]. DMEM without phenol red was used for melanin quantification to prevent any interference with melanin absorbance measurements [47].

20.000 cells were seeded in 6-well plate in MEM. The following day, media was changed to white DMEM. Cells were cultured for 5 days and then harvested to analyze the level of both intra and extracellular melanin, for Immuno Blot analyses, qRT-PCR, PLA, CO-IP, cytosolic-nuclear fractionation, and TEM imaging.

For the pharmacological induction of B16F10 pigmentation, the following compounds were obtained from Sigma-Aldrich: α-Melanocyte stimulating hormone (α − MSH) and CHIR99021. α − MSH is a hormone that stimulates the synthesis of melanin in B16F10 murine melanoma models [47]. A stock solution of 0.5 mM of α-MSH was prepared in deionized water, and then diluted in a phenol red-free cell culture medium to final concentration of 100 nM. Melanoma cells were incubated with α-MSH for 96 h. CHIR99021 is a well-established Wnt signaling activator that acts by inhibiting GSK3-b activity [79], hence favoring b-catenin stabilization which ultimately regulates MITF. CHIR99021 was dissolved in DMSO, and cells were treated with 3 µM CHIR99021 for 48 h. DMSO 0.1% was used as control treatment.

### Measurement of the intracellular and extracellular amount of melanin

According to Chung et al., 2019 [47] for the intracellular melanin content measurement from a cell pellet, 100 μL of 1 N NaOH containing 10% DMSO was added to the pellet and heated at 80 °C for 90 min. Absorbance was then measured at 490 nm using a Tecan Infinite F200PRO micro-plate reader (TECAN). To convert the absorbance value to the amount of melanin, a standard curve was obtained from 0 to 500 μg/mL of synthetic melanin (Cat# 8049-97-6, Sigma-Aldrich) solution dissolved in 1 N NH4OH. For the extracellular melanin quantification, 200 μL of the cell culture medium was transferred to a 96-well plate and the absorbance was read. The absorbance was averaged from three wells, and each experiment was performed in duplicate or triplicate.

### Electron microscopy images to quantify the amount of intracellular melanin granules

Cells grown in 24-wells plates were fixed for 1 h at 4 °C with freshly prepared 2.5% (V/V) glutaraldehyde in 0.1 M sodium cacodylate, pH 7.4. After washing with 0.1 M sodium cacodylate, cells were post-fixed in 1% OsO4, 1.5% K4Fe(CN)6 in 0.1 M sodium cacodylate pH 7.4, stained with 0.5% uranyl acetate, dehydrated in ethanol and embedded in Embed 812. For the samples that followed pigmentation induction, see the relative description above. Thin sections were imaged on a Tecnai-12 electron microscope (Philips-FEI) equipped with a Veleta (Olympus Imaging System) digital camera at the BioImaging Facility of the Dept. of Biology (University of Padua). The experiment was repeated three times. The number of intracellular melanin granules of 15 distinct cells was counted for each biological replicate.

### SDS-PAGE and immunoblot analysis

To obtain cell lysates, freshly harvested cells were washed in 1X PBS, detached and centrifuges 500 *g* for 5 min. To extract whole-cell protein lysates, cold Lysis Buffer was added (20 mM Tris-HCl pH 7.4, 1% Triton X-100, 150 mM NaCl) supplemented with 100X Phosphatase inhibitor cocktails 2 and 3 (P5726-1ml, Sigma; P0044-5ml, Sigma) and 100X Protease inhibitor (P8340-1ml, Sigma). After 30”on/off of pulse sonication at high power using the Branson 250 standard sonifier (Branson), proteins were quantified using BCA assays (Bicinchoninic Acid Assay kit, Cat# 23225, Thermo Fisher Scientific). 50 µg of protein samples were loaded on a 4–12% SDS-PAGE gel and transferred using nitrocellulose membranes in wet condition (Transfer buffer: Tris Glycine 1x, 20% methanol, no SDS). Membranes were later blocked with 5% dried milk powder for 20 min followed by 5% BSA for 40 min, both resuspended in Tris-buffered saline (TBS) and probed with the primary antibodies reported in Table 4. Incubation with primary antibodies was performed over-night at +4 °C. Then, membranes were incubated with a goat anti-rabbit (1:10000) or goat anti-mouse (1:5000) antibody conjugated to horseradish peroxidase (both from Biorad) for 1 h at room temperature, and protein bands were visualized with ECL (Clarity Western ECL Substrate, BioRad). Immunodetection was performed using the ChemiDoc MP Imaging System (BioRad). The uncropped western blots are shown as Supplementary Material.

### Immunoblot densitometry

The software ImageJ was used to analyze the profiles of each lane for the blotted nitrocellulose membrane. The size of the lane selection tool was 8 pixels wide. The lanes’ shapes were represented as the average of the grayscale values or the uncalibrated optical density along a one‐pixel‐height horizontal lane. Protein intensity was calculated as a function of the HRP-band signal. Enrichments in percentage were assigned by normalizing on the housekeeping protein (vinculin, *β*-actin, GAPDH, or lamin A/C), and then on reference control sample.

### Cytosolic-nuclear fractionation

B16F10 cells were rinsed in ice‐cold PBS and collected in lysis buffer containing 20 mM Tris–HCl pH 7.4, 150 mM NaCl, and 1% Triton X‐100 with protease inhibitor cocktail. Nuclear and cytosolic extracts were obtained using the NE‐PER Nuclear and Cytoplasmic Extraction Kit (Thermo Fisher Scientific, Cat# 78833). Protein concentrations were determined by the BCA, using bovine serum albumin as a standard. 20 µg of protein extracts from the different conditions were resolved on sodium dodecyl sulfate (SDS)–polyacrylamide gel and transferred to a nitrocellulose membrane, as previously described.

### Co-immunoprecipitation

After performing cyto-nuclear fractionation, an amount of 700 µg of proteins from the nuclear and the cytosolic extracts of the different conditions were subjected to immunoprecipitation using 6 μg of specific antibodies in combination with 20 μl of Dynabeads™ Protein G (Invitrogen), according to the manufacturer’s instructions. LDS sample buffer 4× (Life Technologies) containing 2.86 M 2‐mercaptoethanol (Sigma‐Aldrich) was added to beads, and samples were boiled at 95 °C for 10 min. Supernatants were analyzed by immuno blot.

### Proximity ligation assay (PLA)

To perform the Proximity Ligation Assay, the Duolink® In Situ Red Mouse/Rabbit kit (DUO92101) from Sigma-Aldrich was employed. Briefly, 15.000 B16F10 WT and TG2 KO cells were plated on 13 mm diameter coverslips. After 48 h, cells were fixed with 4% Paraformaldehyde at room temperature for 15 min, washed three times with PBS, and then permeabilized with 0.2% Triton-X in PBS for 15 min. Blocking was performed using 2% of bovine serum albumin (BSA) in PBS for one hour. Anti-TG2 (Rabbit, #3557 Cell signaling Technologies) and Anti-MITF (Mouse, sc-515925, Santa Cruz Biotechnology) were incubated either or together in all conditions at 4 °C overnight. The next day, coverslips were washed one time with PBS and two times with Wash Buffer A. According to the manufacturer instructions, PLA probes (both MINUS and PLUS) were then diluted in the Antibody diluent and placed on the slides into humidification chamber for 1 h at 37 °C. Ligation was later performed by adding Ligase into the ligation solution buffer and using the slides in a pre-heated humidity chamber for 30 min at +37 °C. After the amplification and probing processes (100 min at 37 °C), slides were later washed five times with PBS, Wash Buffer A and Wash Buffer B and prepared for Imaging. DAPI was used to stain nuclei. Cells were imaged by placing the slides on the stage of a LSM700 (Zeiss) confocal microscopy equipped with a 63X, Zeiss Plan-Apochromat 63x/1.4 oil objective and excited using the appropriate laser line. Images were acquired using a 1048 × 1048 resolution with the ZEN software (Zeiss).

### Real-time reverse transcription PCR (RT-qPCR)

RNA was extracted from cells using 1 ml of PRImeZOL (Canvax, AN1100), according to the manufacturer‟s guidelines. RNAs were then quantified using the Nanodrop spectrophotometer ND-1000 (Thermo Fisher Scientific, Waltham, MA) to calculate the RNA concentration in microliter order. DNase Treatment was later performed to digest the contaminant genomic DNA. The reaction was carried out taking advantage of the DNAse free-kit (Ambion – Life Technologies) using 1 µL recombinant DNAse I and 5 µg of RNA. The reaction was conducted at 37 °C for 30 min. The recombinant DNase I was later inactivated with the DNase Inactivation Reagent (0.1 volume). cDNA was obtained using the SuperScript IV Reverse Transcriptase (Thermofisher, #18091050) following manufacturer’s instructions. 2 µg of RNA was retrotranscribed for each reaction according to the manufacturer‟s instructions. cDNA was diluted and amplified in qRT-PCR using SYBR Green PCR Master Mix (Thermofisher, #4309155). Actin was used as housekeeping control. CT values were first normalized with respect to the housekeeping genes (ΔCT) and next compared to the control sample (ΔΔCT). This relative normalized expression is indicated in the figures. No template controls were used to detect any non-specific amplification. The sequences of RT-qPCR primers are reported in Table 5 for mouse melanoma cells and in Table 6 for human melanoma cell lines.

**Table 5:** Primer used for qRT-PCR analyses of mouse melanoma cells.

mm_Actin_FOR	AGTGTGACGTTGACARCCGT
mm_Actin_REV	TGCTAGGAGCCAGAGCCGTA
mm_AXL_FOR	GGACACCCCCGAGGTACTTA
mm_AXL_REV	GCCGAGGTATAGGCTGTCAC
mm_CITED1_FOR	CCGTACCTCAGCTCCTGTG
mm_CITED1_REV	AGCTGGGCCTGTTGGTCT
mm_Fibronectin1_FOR	ACCATCTACGTCATTGCCCT
mm_Fibronectin1_REV	TTGGGGAAGCTCATCTGTCTT
mm_IGFR1**_**FOR	ACAACTACTGCTCCAAAGACAAA
mm_IGFR1_REV	ATGGCCCTTTATCACCACCA
mm_MCAM_FOR	CGGGTGTGCCAGGAGAG
mm_MCAM_REV	CCACACTTGAGAAGGGCTGT
mm_MITF_FOR	CAACCTCTGAAGAGCAGCAGTT
mm_MITF_REV	GGCGTAGCAAGATGCGTGAT
mm_SPARC_FOR	CTTGGTGGCAAAGAAGTGGC
mm_SPARC_REV	CTTGGTGGCAAAGAAGTGGC
mm_TG2_FOR	GGAGGAGCGACGGGAATATG
mm_TG2_REV	ATTCCATCCTCGAACTGCCC
mm_Tyrosinase_FOR	TCTTCTCCTCCTGGCAGATCA
mm_Tyrosinase_REV	CCTCAGGTGTTCCATCGCAT

**Table 6:** Primer used for qRT-PCR analyses of human melanoma cell lines.

hs_Actin_FOR	GAGCACAGAGCCTCGCCTTT
hs_Actin_REV	TCATCATCCATGGTGAGCTGG
hs_TYRP1_FOR	CCGAAACACAGTGGAAGGTT
hs_TYRP1_REV	TCTGTGAAGGTGTGCAGGA
hs_DCT_FOR	TTTGGTGGGGGTTTCTGCTC
hs_DCT_REV	TATAGCCGGCAAAGTTTCCTGT
hs_MITF_FOR	GGCTTGATGGATCCTGCTTTGC
hs_MITF_REV	GAAGGTTGGCTGGACAGGAGTT
hs_TG2_FOR	CAGGAGAAGAGCGAAGGGAC
hs_TG2_REV	AAGACAAAGGGCGCATCGTA

### Proliferation and colony formation assay

To assess cell proliferation, B16F10 wild-type cells and the two TG2 KO clones were seeded in MEM medium in 96-well plates (2.000 cells/well) and let grow under conditions of 5% CO2 and 37 °C. Cells were blocked at different time points (6 h, 24 h, 48 h, 72 h respectively) to stop and monitor their growth. The wells were washed 3X with PBS and fixed with 4% paraformaldehyde (PFA), then stained with 0.1% Crystal Violet for 30 min and washed multiple times with ddH2O to remove the excess color. The plate’s absorbances were read at 595 nm the Tecan Infinite F2PRO micro plate reader (TECAN). Data collected were analyzed on Excel software. Samples absorbances were normalized on the 6 h condition and then on the control sample.

For colony formation, 500 B16 F10 cells were seeded in a 6-well plate and allowed to grow for 6 days in standard culture medium. The medium was removed, cells were washed twice with PBS and fixed with 3,8% paraformaldehyde for 30 min. After 3 washes with PBS, cells were stained with 0.1% Crystal Violet for 15 min at room temperature and washed with PBS until colonies cleared. Images were taken with Leica MZ stereo microscope16 F with 1 × 0,5 magnification. The number and dimension of colonies were quantified with the ImageJ software.

### siRNA in human melanoma cell lines

The three human melanoma cell lines were knocked down by means of a small interfering RNA (siRNA) silencing technique. siRNA constructs were purchased from ORIGENE (SR322028A) and the cell lines were transfected with a 10 nM TG2 targeting siRNA using RNAiMAX transfection reagent (Thermofisher scientific, #13778150), according to the manufacturer’s protocols for 6-well plates or 12-well plates format. The sense strand of the Transglutaminase 2 human siRNA Oligo Duplex was AGCAACCUUCUCAUCGAGUACUUCC. Non-targeting siRNA control (Universal Scramble negative control) was used as a negative control at a final concentration of 10 nM and was purchased by ORIGENE (SR30004).

### Orthotopic B16F10 Melanoma Model injection for the generation of primary tumors and lung metastases

Animal experiments were carried out according to the Local Ethics Committee of the University of Padua and the National Agency, and under the supervision of the Central Veterinary Service of the University of Padova (in compliance with Italian law DL 116/92 and further modifications, embodying UE directive 86/609), authorization n. 111/2017-PR. Wild-type mice (12 weeks old) in the C57BL6/J background were kept on a 12 h light/dark cycle at controlled temperature and humidity, with standard food (4RF21, Mucedola Srl, Italy) and water provided ad libitum and environmental enrichments. Sub-confluent wild-type murine melanoma B16F10 and clones B16F10 TG2 KO 1 and 2 (70% confluence) were trypsinized, washed and resuspended in PBS. For the primary tumors formation, cell suspension (5 × 10^4^ cells in 100 µl PBS) was injected subcutaneously into the right flank of each mouse. The tumor growth of wild-type and KO clones was assessed by measuring the length and width of each tumor every day and calculating the tumor volume using the formula: TumorVolume = [length ✕ (width)^2^] ✕ 0.5. Fifteen days after tumor cell injection when the tumors impacted on the life quality of the mice, they were euthanized, and their tumors were weighted and harvested. For the lung experimental metastasis formation, 2 × 10^5^ cells resuspended in 100 µl PBS of B16F10 WT and TG2 KO 2 were injected in the caudal vein of the mice. 21 days after injection, mice were euthanized, and their lungs harvested. The number of experimental metastases was counted at optical microscopy.

### Histological sample preparation

After tissues collection, samples were fixed over-night in formalin (Pierce™ 16% Formaldehyde (w/v), Methanol-free, Cat# 28906) at +4 °C. Then, they were washed three times with PBS and dehydrated (50% EtOH 1 h at room temperature, 70% EtOH over-night at +4 °C, 80% EtOH 1 h at room temperature, 90% EtOH 1 h at room temperature, 100% EtOH 2 h at room temperature, Xylene X-free 2 h at room temperature). Dehydrated samples were then embedded in paraffin and cut in 4 µm-thick slices with a microtome.

### Multiplex Immunofluorescence (mIF)

The Tyramide Signal Amplification (TSA)-based Opal method (Akoya Biosciences) was used for mIF staining on the Leica BOND RX automated immunostainer (Leica Microsystems). Prior to staining, all 4 µm-thick FFPE tissue sections were deparaffinised by baking overnight at 56 °C, soaking in BOND Dewax Solution at 72 °C, and then rehydrating in ethanol. Heat-induced epitope retrieval (HIER) pretreatments were applied using BOND Epitope Retrieval (ER) Solutions citrate-based pH 6.0 ER1 or EDTA-based pH 9.0 ER2 (both Leica Biosystems). Tissue sections were blocked with Normal Goat Serum (Vector Laboratories) for 10 min before applying each primary antibody. A fluorescent singleplex was carried out for melanoma cells biomarker to determine the optimal staining conditions. The rabbit anti-mouse Melan-a (Abcam, clone EPR20380) primary antibody was subsequently added on the slides. The HRP-conjugated secondary antibodies goat anti-rabbit (Vector Laboratories) were incubated as appropriated for 10 min. The TSA-conjugated fluorophore was then added for 10 min. Slides were rinsed with washing buffer after each step. Finally, the spectral DAPI (Akoya Biosciences) was used as nuclear counterstain, and slides were mounted in ProLong Diamond Anti-fade Mountant (Life Technologies).

### Multispectral imaging

Multiplex-stained slides were imaged using the Mantra Quantitative Pathology Workstation 2.0 (Akoya Biosciences). The inForm Image Analysis software (version 2.4.9, Akoya Biosciences) was used to unmix multispectral images using a spectral library built from acquisition of single fluorophore-stained control tissues and containing fluorophores-emitting spectral peaks. A selection of representative multispectral images was used to train the inForm software to create algorithms to apply in the batch analysis of all acquired multispectral images. Whole metastases area was calculated with respect to surrounding healthy lung tissue.

### Preparation and analyses of the epidermal skin of C57BL/6 WT and TG2 KO mice

Skin sample from C57BL/6 WT and TG2 KO mice, were fixed with 10% neutral formalin for 16–24 h at room temperature, dehydrated and embedded in paraffin. For histopathological analysis hematoxylin and eosin (H&E) stained tissue sections (4 μm) were used. Animal experiments were carried out according to the Local Ethics Committee of the University of Rome Tor Vergata and the National Agency, and under the supervision of the Central Veterinary Service of the University of Rome Tor Vergata (in compliance with Italian law DL 116/92 and further modifications, embodying UE directive 86/609), authorization n. 111/2017-PR).

### Zebrafish morpholino injection and pigmentation analysis

WT zebrafish were from the Tübingen (Tü) or AB strains. All transgenic lines were collected from original laboratories, which developed the lines and are currently stabled at the zebrafish facility of the University. Fish housing was carried out at 28.5 °C according to standard rules and procedures (https://zfin.org). All animal manipulation procedures were conducted according to the Local Ethical Committee at the University of Padua and National Agency (Italian Ministry of Health) (Italian Ministry of Health Authorization number 407/2015-PR), and with the supervision of the Central Veterinary Service of the University of Padova (in compliance with Italian Law DL 116/92 and further modifications, embodying UE directive 86/609).

For the morpholino injections, Custom Morpholinos were purchased by Genetools LLC. The sequence used is the following one: Tg2bMO: (5′-CCGATGTCCAGAGCCATGTTTATAA-3′). This morpholino affects the translational start of zebrafish TG2 paralogous genes, as we previously demonstrated in Rossin et al. 2021 [52]. Standard Ctrl Morpholino (CtrlMO) was also purchased by Genetools LLC. Microinjection was performed on randomly separated sibling embryos at one-cell stage, adding ≈12 ng/embryos of morpholino. Morpholinos impeded the translation of the zTg2b transcript, blocking both maternal and zygotic forms. Chorions were manually removed at 24 hpf (hpf: hours post fertilization) and images were acquired at 48 hpf. zTg2b KD was performed by the injection of 0.1 pmol of antisense morpholino for embryo. All images were acquired with a Nikon DS-F12 digital camera, at 3.2X magnification. All images were acquired with the same exposure parameters and processed in silico with Gimp 2.0. ImageJ software was used to count melanophores. A single-embryo representative for the whole population was reported in each picture.

### Quantification and statistical analysis

All statistical analyzes were performed with the GraphPad Prism 8 software. Three or more groups were analyzed with one-way Anova. Two-way ANOVA with Bonferroni’s test was used to compare the data with two variables. Unpaired, Student’s *T*-test was used to compare two groups. Additional statistical details can be found in the figure legends.

### Supplementary information

Supplementary Material

Original Data File

aj-checklist

## Data Availability

RNAseq data have been deposited at SRA and are publicly available as of the date of publication (SubmissionID: SUB12302131, BioProject ID: PRJNA904573). This paper does not report original code. Original western blots, microscopy data and all other data reported in this paper will be shared by the lead contact upon request. Any additional information required to reanalyze the data reported in this paper is available from the corresponding author upon request.
