# Supplementary material for: Transglutaminase Type 2-MITF axis regulates phenotype switching in skin cutaneous melanoma
Source: Cell Death Dis. 2023 Oct 28;14(10):704. doi: 10.1038/s41419-023-06223-y (PMC10613311; doi:10.1038/s41419-023-06223-y)
Supplement: Supplementary file 1 — Supplementary Material [file 41419_2023_6223_MOESM1_ESM.pdf]

SUPPLEMENTARY MATERIAL

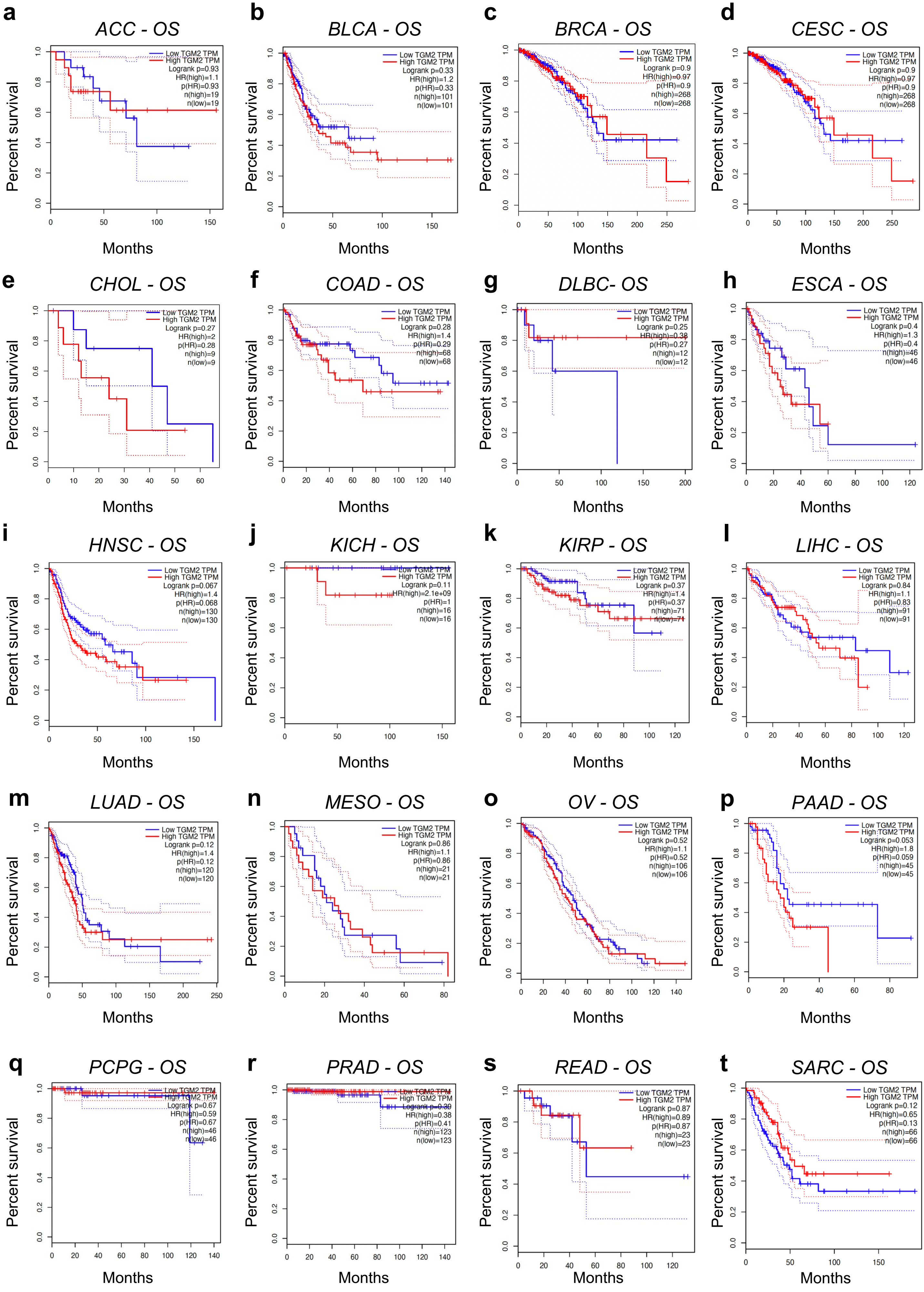
a
ACC - OS
Low TGM2 TPM
High TGM2 TPM
Logrank p=0.93
HR(high)=1.1
p(HR)=0.93
n(high)=19
n(low)=19
b
BLCA - OS
Low TGM2 TPM
High TGM2 TPM
Logrank p=0.33
HR(high)=1.2
p(HR)=0.33
n(high)=101
n(low)=101
c
BRCA - OS
Low TGM2 TPM
High TGM2 TPM
Logrank p=0.9
HR(high)=0.97
p(HR)=0.9
n(high)=268
n(low)=268
d
CESC - OS
Low TGM2 TPM
High TGM2 TPM
Logrank p=0.9
HR(high)=0.97
p(HR)=0.9
n(high)=268
n(low)=268
e
CHOL - OS
Low TGM2 TPM
High TGM2 TPM
Logrank p=0.27
HR(high)=2
p(HR)=0.28
n(high)=9
n(low)=9
f
COAD - OS
Low TGM2 TPM
High TGM2 TPM
Logrank p=0.28
HR(high)=1.4
p(HR)=0.29
n(high)=68
n(low)=68
g
DLBC- OS
Low TGM2 TPM
High TGM2 TPM
Logrank p=0.25
p(HR)=0.27
n(high)=12
n(low)=12
h
ESCA - OS
Low TGM2 TPM
High TGM2 TPM
Logrank p=0.4
HR(high)=1.3
p(HR)=0.4
n(high)=46
n(low)=46
i
HNSC - OS
Low TGM2 TPM
High TGM2 TPM
Logrank p=0.067
HR(high)=1.4
p(HR)=0.068
n(high)=130
n(low)=130
j
KICH - OS
Low TGM2 TPM
High TGM2 TPM
Logrank p=0.11
HR(high)=2.1e+09
p(HR)=1
n(high)=16
n(low)=16
k
KIRP - OS
Low TGM2 TPM
High TGM2 TPM
Logrank p=0.37
HR(high)=1.4
p(HR)=0.37
n(high)=71
n(low)=71
l
LIHC - OS
Low TGM2 TPM
High TGM2 TPM
Logrank p=0.84
HR(high)=1.1
p(HR)=0.83
n(high)=91
n(low)=91
m
LUAD - OS
Low TGM2 TPM
High TGM2 TPM
Logrank p=0.12
HR(high)=1.4
p(HR)=0.12
n(high)=120
n(low)=120
n
MESO - OS
Low TGM2 TPM
High TGM2 TPM
Logrank p=0.86
HR(high)=1.1
p(HR)=0.86
n(high)=21
n(low)=21
o
OV - OS
Low TGM2 TPM
High TGM2 TPM
Logrank p=0.52
HR(high)=1.1
p(HR)=0.52
n(high)=106
n(low)=106
p
PAAD - OS
Low TGM2 TPM
High TGM2 TPM
Logrank p=0.053
HR(high)=1.8
p(HR)=0.059
n(high)=45
n(low)=45
q
PCPG - OS
Low TGM2 TPM
High TGM2 TPM
Logrank p=0.67
HR(high)=0.59
p(HR)=0.67
n(high)=46
n(low)=46
r
PRAD - OS
Low TGM2 TPM
High TGM2 TPM
HR(high)=0.38
p(HR)=0.41
n(high)=123
n(low)=123
s
READ - OS
Low TGM2 TPM
High TGM2 TPM
Logrank p=0.87
HR(high)=0.89
p(HR)=0.87
n(high)=23
n(low)=23
t
SARC - OS
Low TGM2 TPM
High TGM2 TPM
Logrank p=0.12
HR(high)=0.65
p(HR)=0.13
n(high)=66
n(low)=66
Percent survival
Months

Fig. S1a

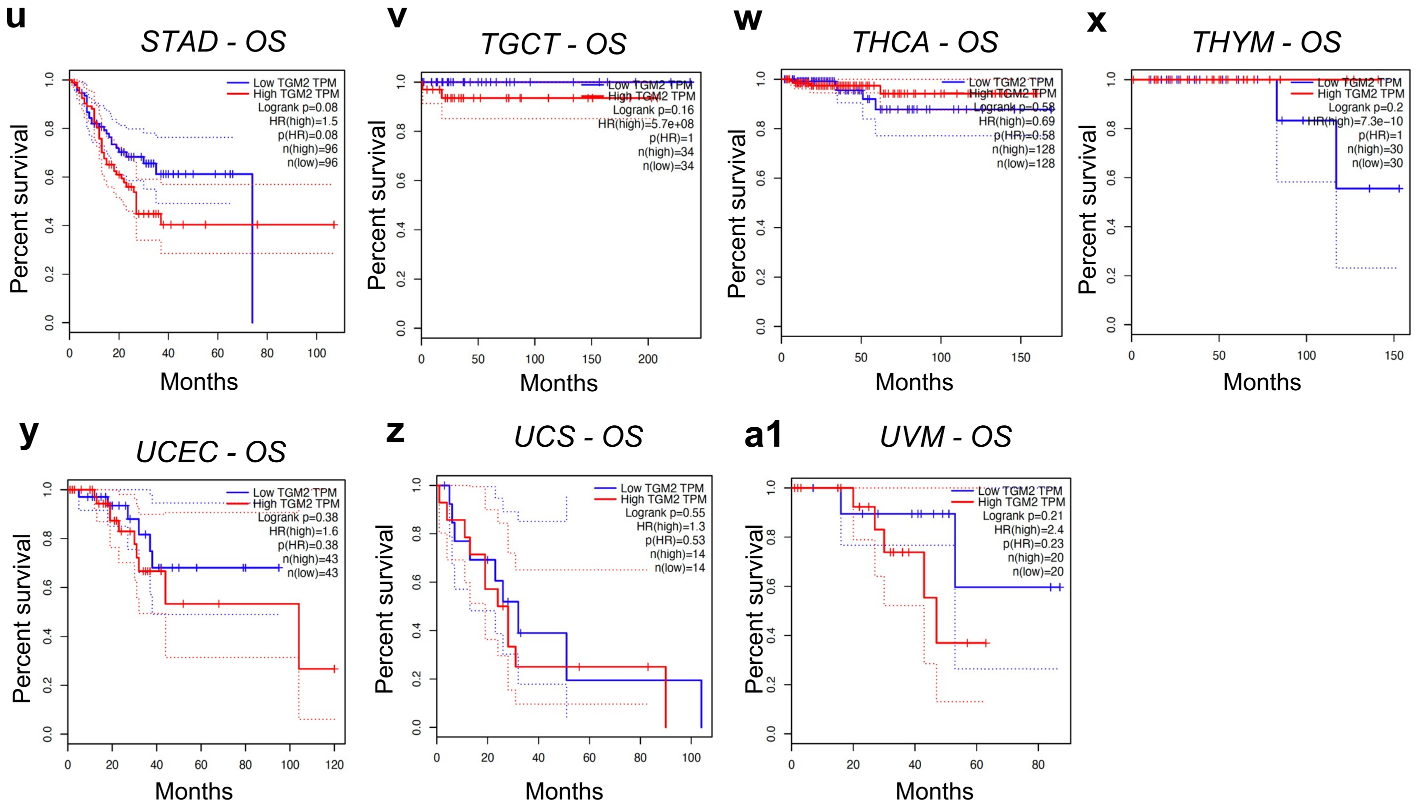
u
STAD - OS
Low TGM2 TPM
High TGM2 TPM
Logrank p=0.08
HR(high)=1.5
p(HR)=0.08
n(high)=96
n(low)=96
Percent survival
Months
v
TGCT - OS
Low TGM2 TPM
High TGM2 TPM
Logrank p=0.16
HR(high)=5.7e+08
p(HR)=1
n(high)=34
n(low)=34
Percent survival
Months
w
THCA - OS
Low TGM2 TPM
High TGM2 TPM
Logrank p=0.58
HR(high)=0.69
p(HR)=0.58
n(high)=128
n(low)=128
Percent survival
Months
x
THYM - OS
Low TGM2 TPM
High TGM2 TPM
Logrank p=0.2
HR(high)=7.3e−10
p(HR)=1
n(high)=30
n(low)=30
Percent survival
Months
y
UCEC - OS
Low TGM2 TPM
High TGM2 TPM
Logrank p=0.38
HR(high)=1.6
p(HR)=0.38
n(high)=43
n(low)=43
Percent survival
Months
z
UCS - OS
Low TGM2 TPM
High TGM2 TPM
Logrank p=0.55
HR(high)=1.3
p(HR)=0.53
n(high)=14
n(low)=14
Percent survival
Months
a1
UVM - OS
Low TGM2 TPM
High TGM2 TPM
Logrank p=0.21
HR(high)=2.4
p(HR)=0.23
n(high)=20
n(low)=20
Percent survival
Months

Fig. S1b

a

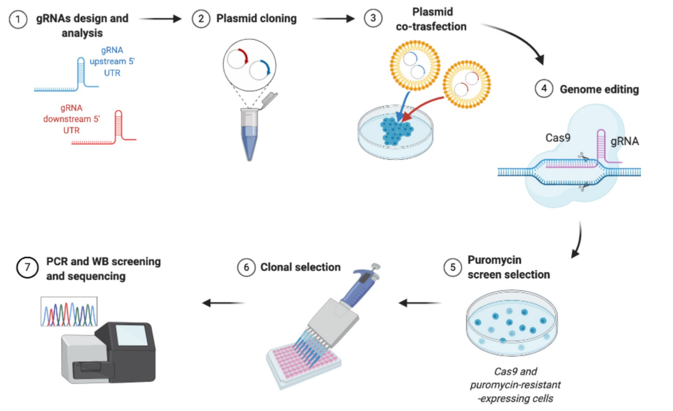

b

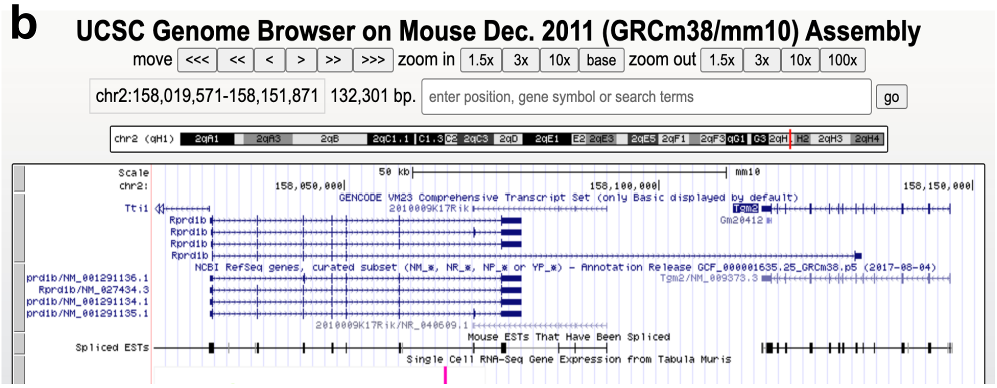

c

guideRNAs couple	Deletion size	Amplicon size
wt	-	1398 bps
1+3	264bps	1134 bps
2+3	397bps	1001 bps

d

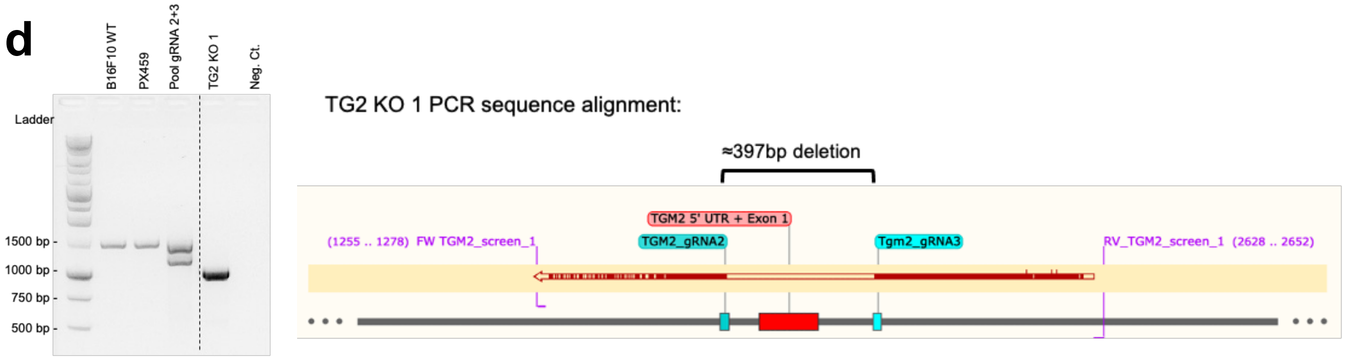

e

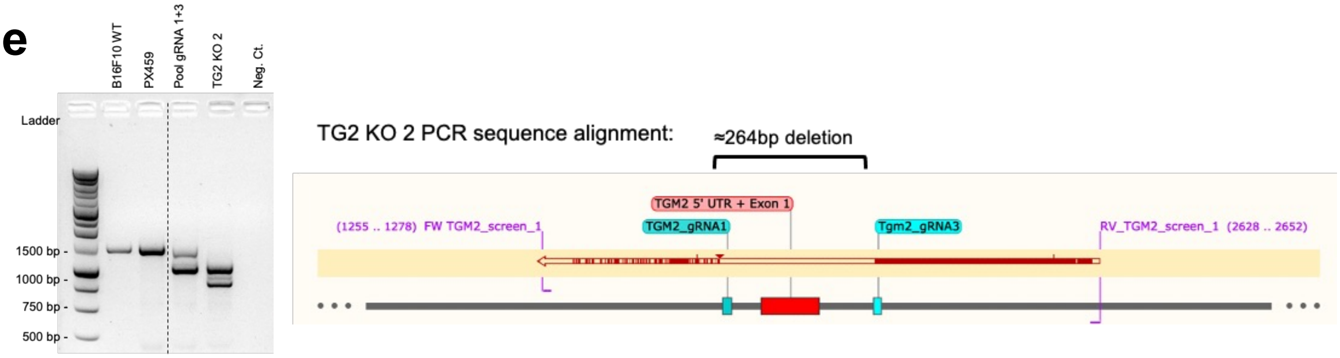

Fig. S2

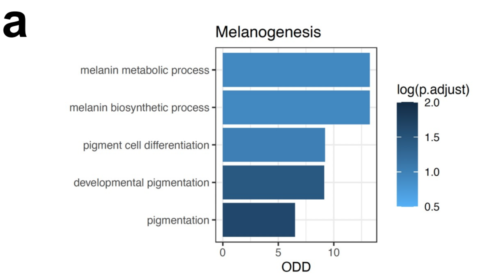

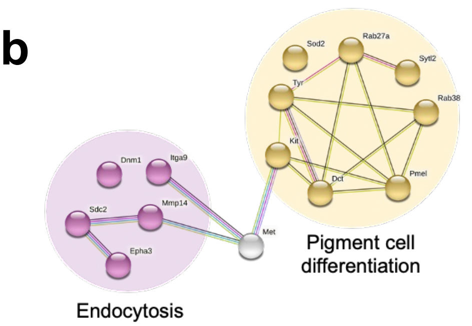

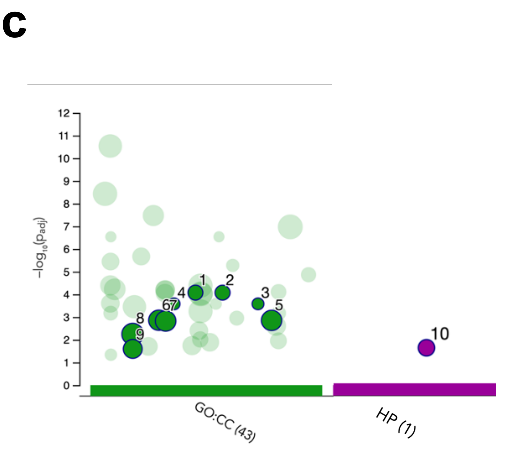

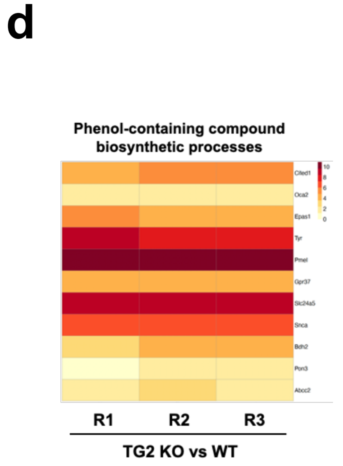

	Source	ID	Name	p_{adj}
1	GO:CC	GO:0042470	Melanosome	8.384×10^{-5}
2	GO:CC	GO:0048770	Pigment granule	8.384×10^{-5}
3	GO:CC	GO:0090741	Pigment granule membrane	2.625×10^{-4}
4	GO:CC	GO:0033162	Melanosome membrane	2.625×10^{-4}
5	GO:CC	GO:0097708	Intracellular vesicle	1.406×10^{-3}
6	GO:CC	GO:0031410	Cytoplasmic vesicle	1.383×10^{-3}
7	GO:CC	GO:0031982	Vesicle	1.494×10^{-3}
8	GO:CC	GO:0012505	Endomembrane system	5.660×10^{-3}
9	GO:CC	GO:0012506	Vesicle membrane	2.542×10^{-2}
10	HP	HP:0005599	Hypopigmentation of Hair	4.104×10^{-2}

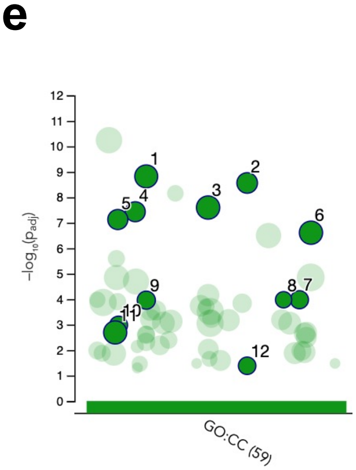

	Source	ID	Name	p_{adj}
1	GO:CC	GO:0030054	Cell junction	1.508×10^{-9}
2	GO:CC	GO:0070161	Anchoring junction	2.736×10^{-9}
3	GO:CC	GO:0042995	Cell projection	2.525×10^{-8}
4	GO:CC	GO:0015629	Actin cytoskeleton	3.692×10^{-8}
5	GO:CC	GO:0005911	Cell-cell junction	7.457×10^{-8}
6	GO:CC	GO:0120025	Plasma membrane bounded cell projection	2.456×10^{-7}
7	GO:CC	GO:0098862	Cluster of actin-based cell projections	1.053×10^{-4}
8	GO:CC	GO:0097517	Contractile actin filament bundle	1.056×10^{-4}
9	GO:CC	GO:0030055	Cell-substrate junction	1.095×10^{-3}
10	GO:CC	GO:0005925	Focal adhesion	1.047×10^{-3}
11	GO:CC	GO:0005856	Cytoskeleton	2.027×10^{-3}
12	GO:CC	GO:0070160	Tight junction	4.126×10^{-2}

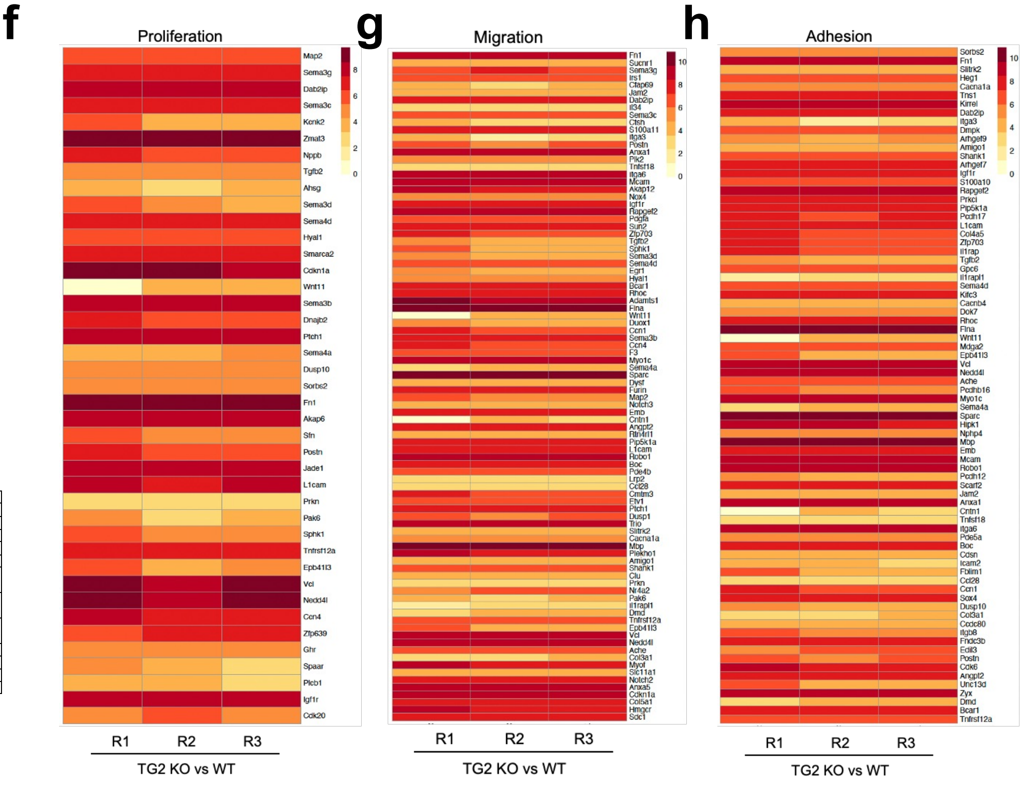

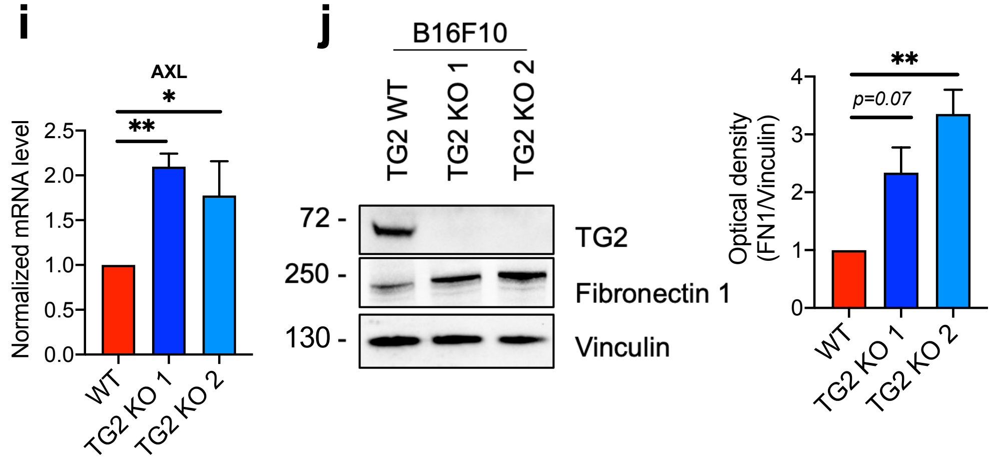

Fig. S3

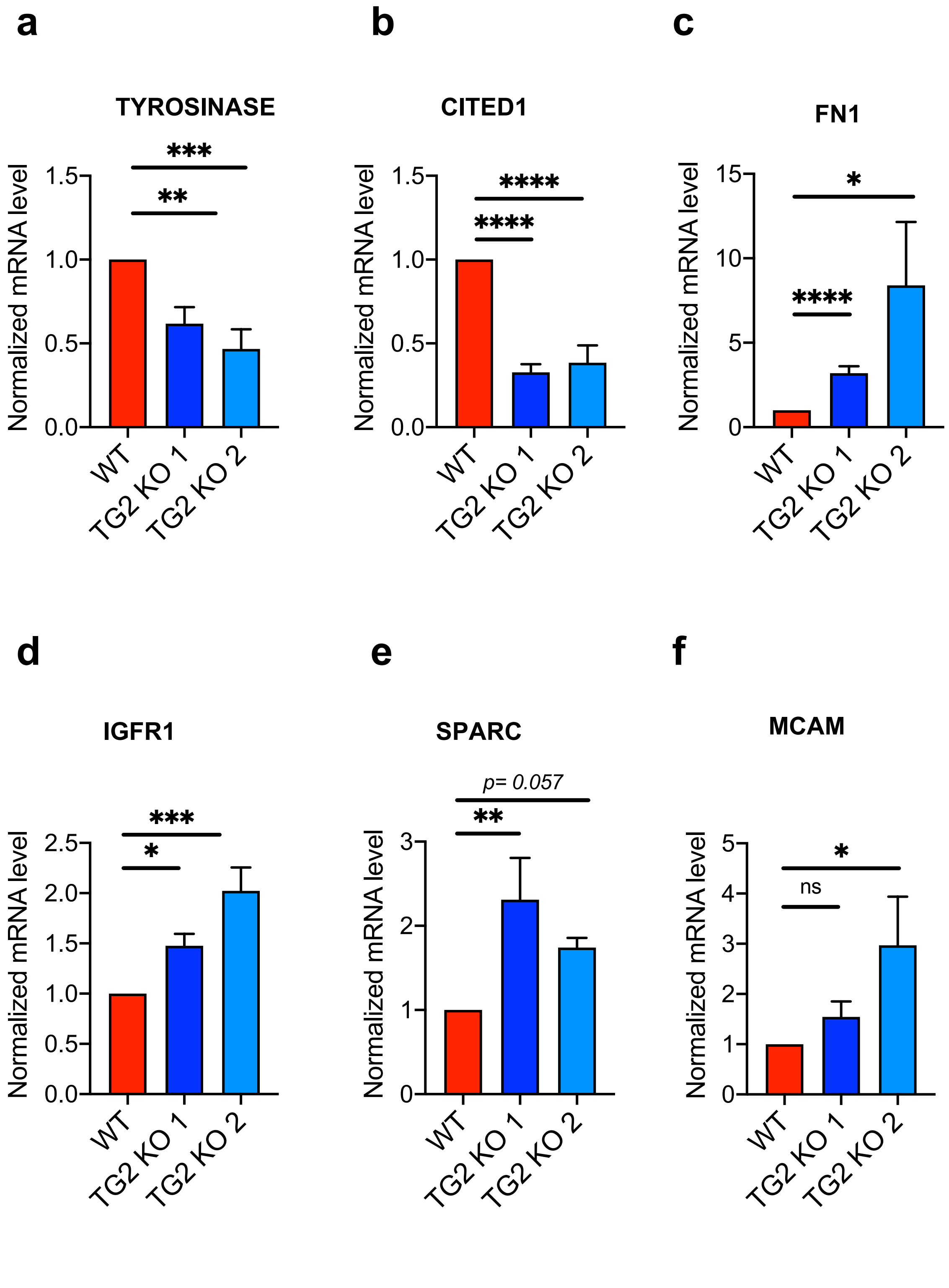
a
TYROSINASE
***
**
Normalized mRNA level
0.0
0.5
1.0
1.5
WT
TG2 KO 1
TG2 KO 2
b
CITED1
****
****
Normalized mRNA level
0.0
0.5
1.0
1.5
WT
TG2 KO 1
TG2 KO 2
c
FN1
*
****
Normalized mRNA level
0
5
10
15
WT
TG2 KO 1
TG2 KO 2
d
IGFR1
***
*
Normalized mRNA level
0.0
0.5
1.0
1.5
2.0
2.5
WT
TG2 KO 1
TG2 KO 2
e
SPARC
p= 0.057
**
Normalized mRNA level
0
1
2
3
WT
TG2 KO 1
TG2 KO 2
f
MCAM
*
ns
Normalized mRNA level
0
1
2
3
4
5
WT
TG2 KO 1
TG2 KO 2

Fig. S4

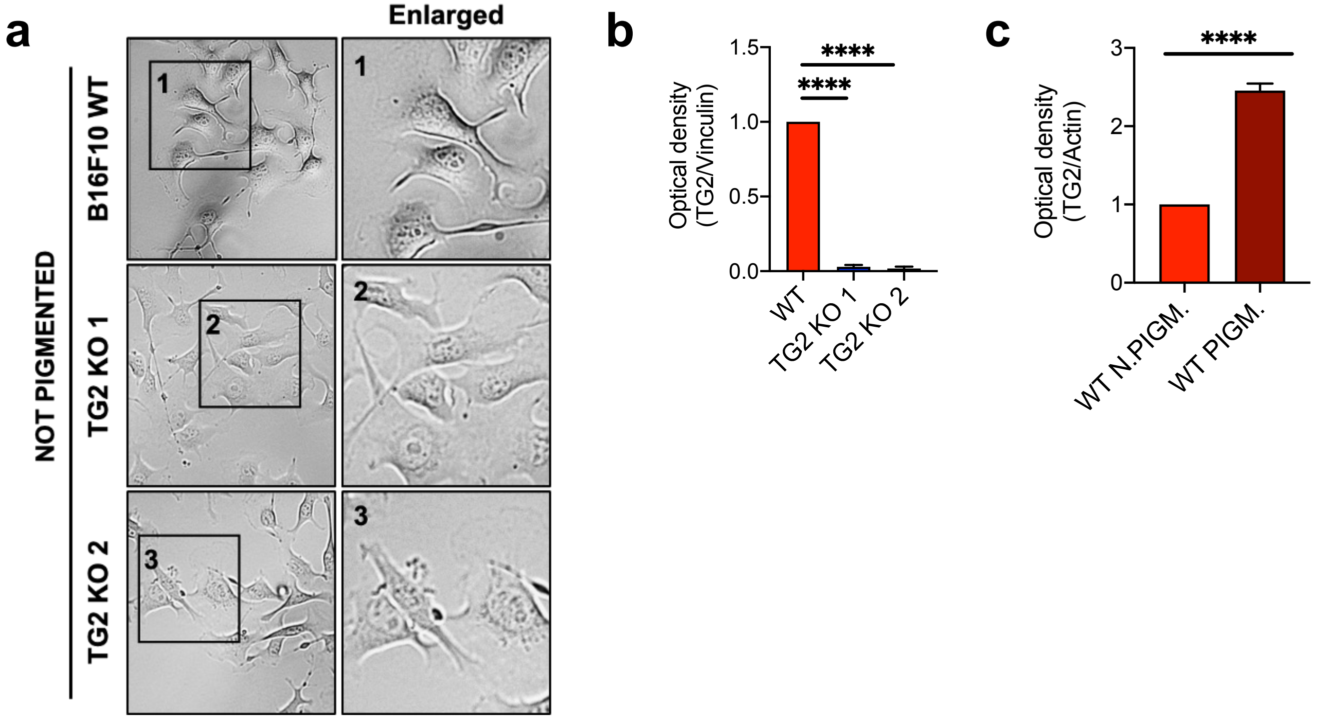
a
Enlarged
NOT PIGMENTED
B16F10 WT
TG2 KO 1
TG2 KO 2
1
2
3
b
Optical density (TG2/Vinculin)
1.5
1.0
0.5
0.0
****
****
WT
TG2 KO 1
TG2 KO 2
c
Optical density (TG2/Actin)
3
2
1
0
****
WT N.PIGM.
WT PIGM.

Fig. S5

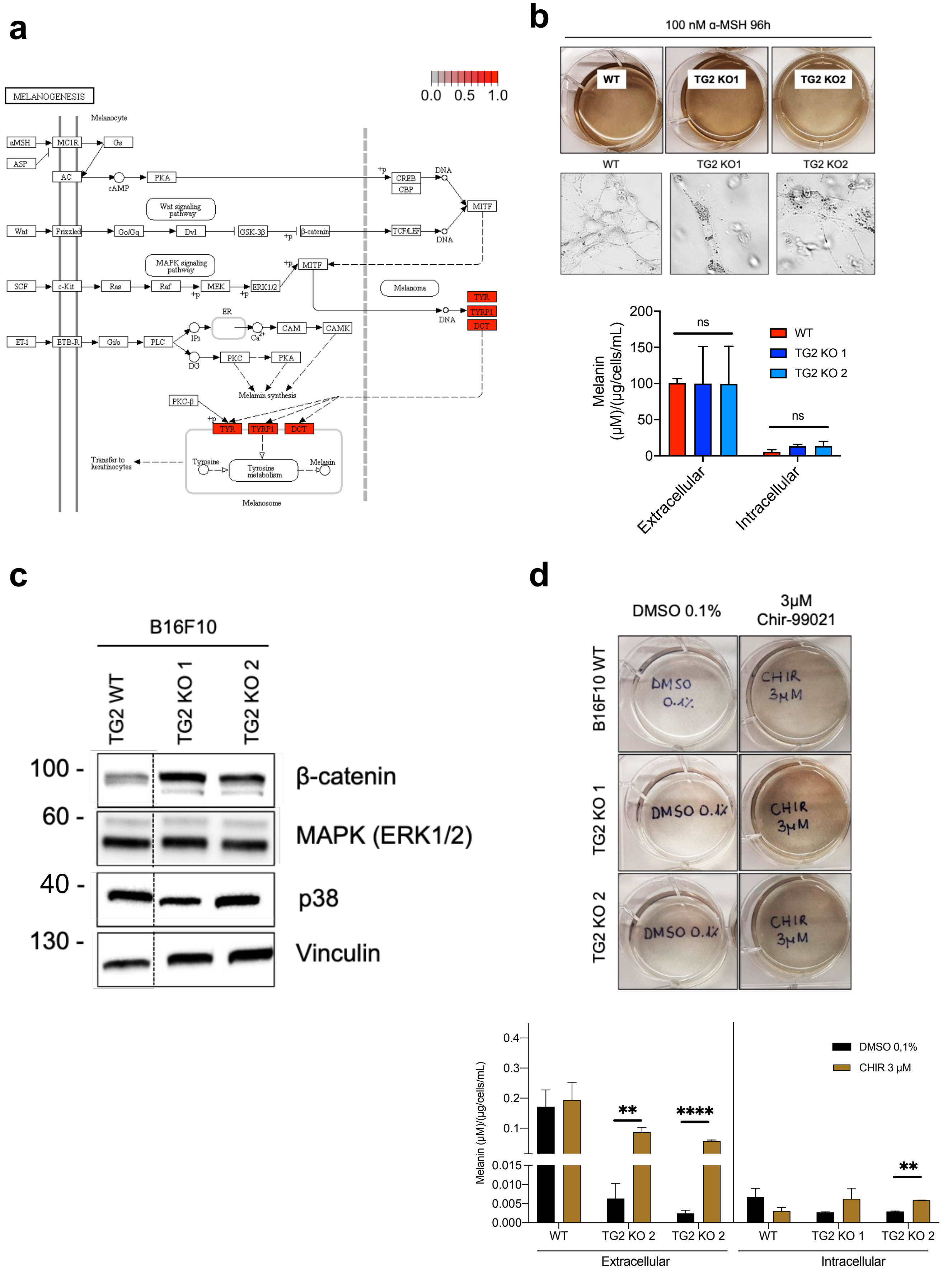
a
MELANOGENESIS
0.0 0.5 1.0
Melanocyte
αMSH
ASP
MC1R
Gs
AC
cAMP
PKA
CREB
CBP
DNA
MITF
Wnt signaling pathway
Wnt
Frizzled
Go/Gq
Dvl
GSK-3β
β-catenin
TCF/LEF
MAPK signaling pathway
SCF
c-Kit
Ras
Raf
MEK
ERK1/2
Melanoma
TYR
TYRP1
DCT
ER
ET-1
ETB-R
Gi/o
PLC
IP3
DG
CAM
CAMK
PKC
Melanin synthesis
PKC-β
Transfer to keratinocytes
Tyrosine
Tyrosine metabolism
Melanin
Melanosome
b
100 nM α-MSH 96h
WT
TG2 KO1
TG2 KO2
Melanin (μM)/(μg/cells/mL)
ns
WT
TG2 KO 1
TG2 KO 2
Extracellular
Intracellular
c
B16F10
TG2 WT
TG2 KO 1
TG2 KO 2
100 -
60 -
40 -
130 -
β-catenin
MAPK (ERK1/2)
p38
Vinculin
d
DMSO 0.1%
3μM Chir-99021
B16F10 WT
TG2 KO 1
TG2 KO 2
Melanin (μM)/(μg/cells/mL)
DMSO 0,1%
CHIR 3 μM
WT
TG2 KO 2
TG2 KO 2
WT
TG2 KO 1
TG2 KO 2
Extracellular
Intracellular

Fig. S6

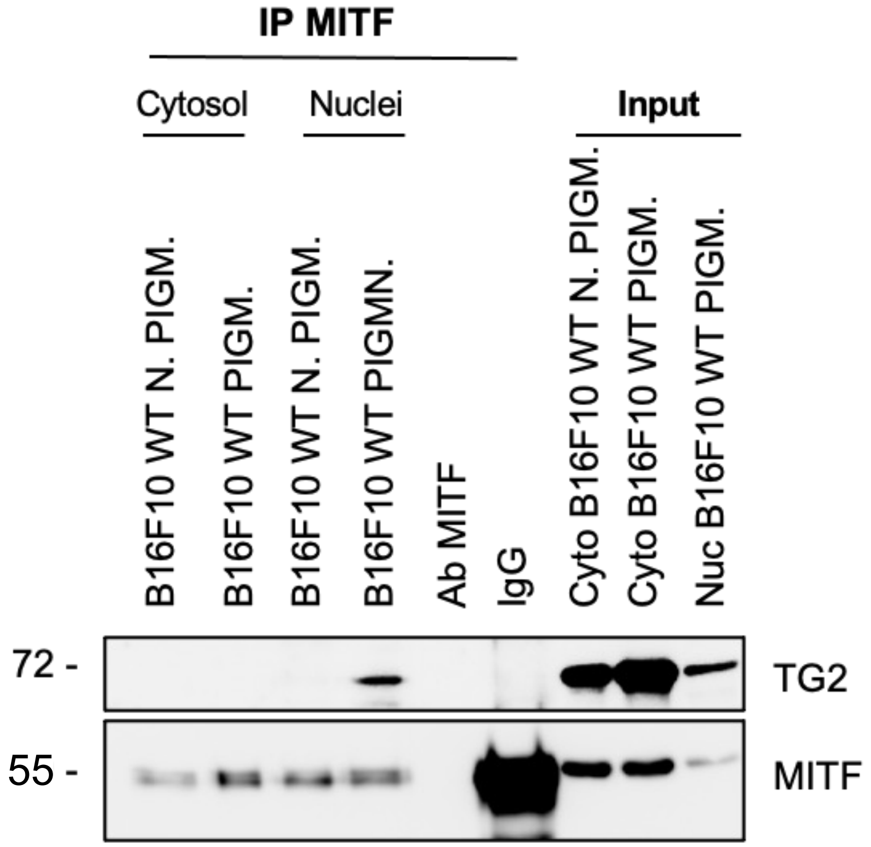
IP MITF
Cytosol
Nuclei
Input
B16F10 WT N. PIGM.
B16F10 WT PIGM.
B16F10 WT N. PIGM.
B16F10 WT PIGMN.
Ab MITF
IgG
Cyto B16F10 WT N. PIGM.
Cyto B16F10 WT PIGM.
Nuc B16F10 WT PIGM.
72 -
55 -
TG2
MITF

Fig. S7

a

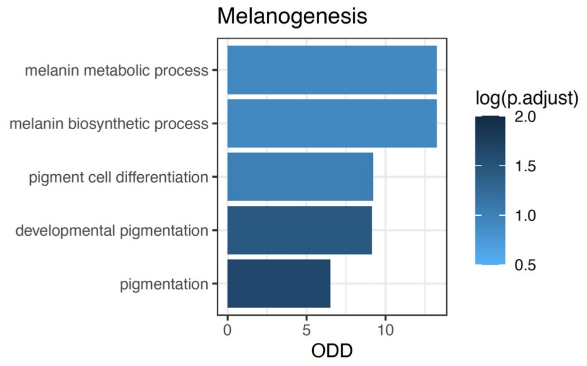

b

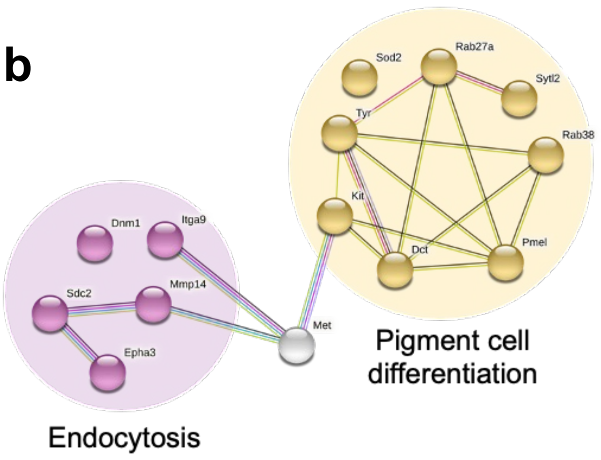

c

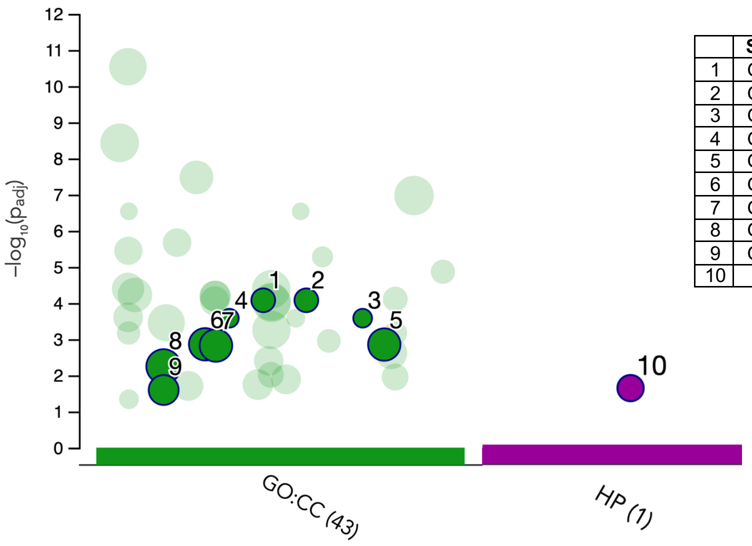

	Source	ID	Name	p_{adj}
1	GO:CC	GO:0042470	Melanosome	8.384×10^{-5}
2	GO:CC	GO:0048770	Pigment granule	8.384×10^{-5}
3	GO:CC	GO:0090741	Pigment granule membrane	2.625×10^{-4}
4	GO:CC	GO:0033162	Melanosome membrane	2.625×10^{-4}
5	GO:CC	GO:0097708	Intracellular vesicle	1.406×10^{-3}
6	GO:CC	GO:0031410	Cytoplasmic vesicle	1.383×10^{-3}
7	GO:CC	GO:0031982	Vesicle	1.494×10^{-3}
8	GO:CC	GO:0012505	Endomembrane system	5.660×10^{-3}
9	GO:CC	GO:0012506	Vesicle membrane	2.542×10^{-2}
10	HP	HP:0005599	Hypopigmentation of Hair	4.104×10^{-2}

d

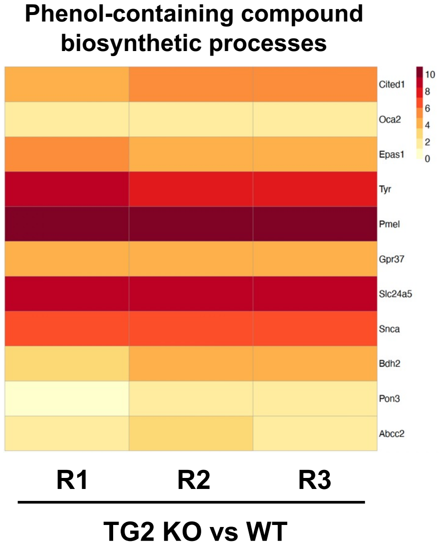

Fig. S3A

Supplementary Figure Legends:

Fig. S1: Analysis of the TG2 clinical value in TCGA cancer datasets. a-a1) Overall survival based on TG2 expression level in TCGA cancer datasets was obtained through Kaplan-Meier analysis by sorting samples for high and low TG2 expression groups according to the quartile (Cutoff-High= 25%; Cutoff-Low= 75%) on GEPIA. Percent survival was plotted, and *p*-values were shown as per figure specification, respectively. Cancer names list: ACC: Adrenocortical carcinoma; BLCA: Bladder Urothelial Carcinoma; BRCA: Breast invasive carcinoma; CESC: Cervical squamous cell carcinoma and endocervical adenocarcinoma; CHOL: Cholangio carcinoma; COAD: Colon adenocarcinoma; DLBC: Lymphoid Neoplasm Diffuse Large B-cell Lymphoma; ESCA: Esophageal carcinoma; HNSC: Head and Neck squamous cell carcinoma; KICH: Kidney Chromophobe; KIRP: Kidney renal papillary cell carcinoma; LAML: Acute Myeloid Leukemia; LIHC: Liver hepatocellular carcinoma; LUAD: Lung adenocarcinoma; MESO: Mesothelioma; OV: Ovarian serous cystadenocarcinoma; PAAD: Pancreatic adenocarcinoma; PCPG: Pheochromocytoma and Paraganglioma; PRAD: Prostate adenocarcinoma; READ: Rectum adenocarcinoma; SARC: Sarcoma; STAD: Stomach adenocarcinoma; TGCT: Testicular Germ Cell Tumors; THCA: Thyroid carcinoma; THYM: Thymoma; UCEC: Uterine Corpus Endometrial Carcinoma; UCS: Uterine Carcinosarcoma; UVM: Uveal Melanoma.

Fig. S2: Generation of TG2 KO B16F10 melanoma clones. a) Schematic view of the strategy used. Briefly, after the design and bioinformatic validations (1), sgRNAs were cloned in the PX459 plasmids carrying the coding sequence for the Cas9 protein and a puromycin resistance cassette (2). Vectors carrying the sgRNAs were co-transfected (one vector carrying the sgRNA mapping upstream and the other mapping downstream the targeted sequence) in B16F10 WT cells (3) to let genomic editing occur (4). After puromycin selection (5) and single cells seeding (6), single clones were validated by PCR, immunoblot, and qRT-PCR analyses (7). **b)** *TGM2* genomic sequence

visualization on UCSC Genome Browser (*https://genome.ucsc.edu/*). The 3' terminus of the *TGM2* gene is partially overlapping with one of the isoforms of *RPRD1B* gene. Therefore, to avoid any off-target effect during the CRISPR/Cas9 genetic editing, the deletion covered only the 5' end of the *TGM2* gene, to prevent both the initiation of transcription and translation of TG2. **c)** Table representing the deletion size of the CRISPR/Cas9 genome editing on the 5' end of *TGM2* and the expected amplicon size relative to the validation PCR, depending on sgRNAs pair. Primer pairs were designed to both induce a deletion that covers the *TGM2* transcription initiation site (TSS), the translation initiation site, and the promoter region. Total WT amplicon size is expected to be around 1,400 bps. sgRNA pair 1+3 leads to a deletion of about 160 bps (expected amplicon size: around 1,100 bps). sgRNA pair 2+3 leads to a deletion of around 400 bps (expected amplicon size around 1,000 bps). **d-e)** PCR validations performed with the FW TGM2_screen_1 and RV TGM2_screen_1 primers on the B16F10 clone TG2 KO 1 (D) and TG2 KO 2 (E). Amplicons were sequenced to check the deletion with respect to the WT cell lines.

Fig. S3: Multi-omics analyses on the downregulated and upregulated GO of Biological Processes (BPs) in TG2 KO clones. a) Bar plot representative of the GO enrichment analyses of the melanogenesis-related Biological Processes (BPs). Bar color represents the *adj.p-value* (dark blue= most significant). Bar lengths refer to the proportion of enriched proteins for each term. **b)** STRING (Search Tool for the Retrieval of Interacting Genes) plot showing two protein-protein interaction clusters using the downregulated TG2 KO targets that are involved in endocytosis and pigment cell differentiation. Clustering has been performed using the ClusterONE App on Cytoscape 3.9.1. **c)** Analyses of the GO of Cellular Components (CCs) and Human Pathologies (HP) retrieved from gProfiler using the downregulated target proteins in TG2 KOs, in green and purple respectively. Dot size represent the protein enrichment for each term. Selected dots represent GOs involved in the process of pigmentation. The complete list of highlighted GOs is reported in the table on the right. **d)**

Heat map of comparative differentially expressed genes (DEGs) of the phenol-containing compound biosynthetic processes mRNA targets, generated using pheatmap R package. Three biological replicates of TG2 KO 2 were subjected to RNAseq analysis. Significant downregulated transcripts are represented according to the *adj.p-value* (dark red= more significantly downregulated). **e)** Analyses of the GO of Cellular Components (CCs) retrieved from gProfiler using the upregulated target proteins in TG2 KOs. Dot size represent the protein enrichment for each term. Selected dots represent GOs involved in the process migration and cell adhesion. The complete list of highlighted GOs is reported in the table on the right. **f-h)** Heat map of comparative differentially expressed genes (DEGs) of the Proliferation (B), Migration (C), and Adhesion (D) RNA target categories, generated using pheatmap R package. Three biological replicates of TG2 KO 2 were subjected to RNAseq analysis. Significant upregulated transcripts are represented according to the *adj.p-value* (dark red= more significantly upregulated). **i)** AXL gene expression evaluated by qRT-PCR analysis in B16F10 WT cells and TG2 KO clones (n=3). *β*-actin was used as housekeeping gene for the normalization. **j)** Immunoblot analyses and relative densitometry of Fibronectin 1 (FN1) expression in B16F10 WT, TG2 KO 1 and TG2 KO 2 cells. Vinculin was used as loading control (n=3). Statistical significance was calculated with One-Way ANOVA and specified with asterisks (*$p < 0.05$, **$p < 0.01$, ***$p < 0.001$, ****$p<0.0001$). Data are represented as mean ± SEM.

Fig. S4: qRT-PCR validations based on RNAseq identified up and downregulated targets. a-f) Tyrosinase (A), Cited1 (B), Fibronect1 (FN1) (C), IGRF1 (D), Sparc (E), and Mcam (F) gene expression evaluated by qRT-PCR in B16F10 WT cells and TG2 KO clones (n=3). Statistical significance was calculated with One-Way ANOVA and specified with asterisks (*$p < 0.05$, **$p < 0.01$, ***$p < 0.001$, ****$p<0.0001$). Data are represented as mean ± SEM.

Fig. S5: TG2 is required during melanogenesis: a) Morphological analysis of B16F10 WT and TG2 KO clones in normal conditions. Cellular shape is enlarged. Cells appear round shaped. No significant difference between the samples is appreciable. Scale bar = 200 μm. **b-c)** Immunoblot densitometric analyses showing the expression of TG2 in WT, TG2 KO 1, and TG2 KO 2 in normal conditions and TG2 expression in B16F10 WT cells following or not pigmentation. Vinculin was used as loading control (n=5). Statistical significance was calculated with One-Way ANOVA and specified with asterisks (*$p < 0.05$, **$p < 0.01$, ***$p < 0.001$, ****$p<0.0001$). Data are represented as mean ± SEM.

Fig. S6: TG2 is not involved in MITF canonical activating pathways. a) KEGG pathway showing the known interactions within melanogenesis related proteins. Significantly downregulated proteins from the Proteomics analysis of the TG2 KO clones are reported in red. **b)** Analysis of the effect of 96 hours 100 nM α-MSH treatment on TG2 KO clones versus WT cell line and relative melanin quantification. After treatment with α-MSH, all the samples released melanin (brown color) in the growing media. Also, cells display the typical differentiated dendritic shape, as well as melanin granules enriched in the cytosol. Extracellular and intracellular melanin levels were quantified and expressed in (μM)/(μg/cells/mL). Analyses were normalized on the cell mass. B16F10 WT was used as control during statistical analysis (n=6). **c)** Immunoblot analysis showing the expression of β-catenin, MAPK (ERK1/2), and p38 in WT, TG2 KO 1, and TG2 KO 2 in normal conditions and TG2 expression in B16F10 WT cells following or not pigmentation. Vinculin was used as loading control (n=3). **d)** Analysis of the effect of 24 hours 3 μM CHIR-99021, a Wnt activator, on TG2 KO clones versus WT cell line and relative melanin quantification. After treatment with CHIR, all the samples released melanin (brown color) in the growing media. Extracellular and intracellular melanin levels were quantified and expressed in (μM)/(μg/cells/mL). Analyses were normalized on the cell mass. B16F10 WT was used as control during statistical analysis (n=6). Statistical significance was

calculated with One-Way ANOVA and specified with asterisks (*$p < 0.05$, **$p < 0.01$, ***$p < 0.001$, ****$p<0.0001$). Data are represented as mean ± SEM.

Fig. S7: Co-IP assay showing the interaction between TG2 and MITF. The interaction is enriched in the nuclei of B16F10 WT pigmenting cells.

Supplementary Tables:

Table 1 - TG2 KO 1 vs WT Proteomics downregulated targets:

Tgm2	Cbr3	Fam213a	Ptgds	Dpysl5	Gfpt2
Sytl2	Cryl1	Gpd1	Kit	Fabp7	Sgsm3
Slc38a2	Cth	Itga9	Uqcrb	C5	Tyr
Dok1	Kmt2b	Hist1h1a	Ppif	Pgm2l1	Arl15
Plin2	Fndc3c1	Dct	C1qtnf3	Ca6	Serpinc1
Mtfp1	Rps27a	Ca14	Comp	Mt-Cyb|mt-Cytb	Rab38
Rab27a	Nup50	Wdhd1	Epha3	Dnajc15	Spry4
Uqcrc1	Scn8a	Q91V76	Sdc1	Nckap5l	Gnl3
Bcat1	Uqcrc2	Nt5c2	Uqcrfs1	Entpd6	Ptpn6
Nufip1	Cox7a2l	Iqgap2	Eef2k	Ldhb	Met
Peg10	Nol12	Sub1	Eif4b	Nudt16l1	Smarcd1
Golm1	Ube2e1	Sdc2	Zcchc7	Uqcrq	Zfp219

Table 2 - TG2 KO 2 vs WT Proteomics downregulated targets:

Tgm2	Pycard	Tyr	Ptgds	Itga9	Fabp7
Fgd2	Pmel	Snca	Iqgap2	Arfgef3	Dpysl5
Cryl1	Akr1c13	Syt4	Gfpt2	Mgst1	Ogdhl
Hsd17b11	Slc37a1	Tmem56	Rab27a	Rap1gap	Ada
Fndc3c1	Kmt2b	Mogat2	Scn8a	Neu1	Adam12
Osbpl6	Mmp14	Fth1	Rab38	Uhrf1	Eef2k
Cyp2j6	Top2a	Sgip1	Eps8	Tpcn1	Dstyk
Cotl1	Dusp4	Rassf2	Agpat4	Smarcd1	Itpk1
Lpcat2	Tubb3	Txnrd3	Igsf8	Cdk2	Sod2
Q91V76	Slirp	Dnm1	Donson	Sytl2	Ehhadh
Oard1	Ube2e1	Telo2	C1qtnf3	Lrp8	Eml4
Ptpn6	Dnph1	Endog	Etl4	Nudt16l1	Cyb5a
Selh	Timeless	Hmcn1	Lrpprc		

Table 3 - TG2 KO 1 vs WT Proteomics upregulated targets:

Ugt1a7c	Gbp2	Epb41l3	S100a10	Steap1	Hoxb9
Anxa2	L1cam	Tppp3	S100a11	Steap2	Anxa1
Nexn	Postn	Ehd2	Pycard	Tagln2	Sparc
Cald1	Fam107b	Cd9	Kank4	Ass1	Smap
Ahnak	Eml1	Map1a	Tpm4	Pcdh7	Parva
Anxa5	Fosl1	Synpo	Sphk1	Nfix	Dclk1
Nqo1	Rcn2	Anxa3	Ecm1	Prkcq	Serpinb9b
Podxl	Tubb3	Cxadr	Capn2	Aox1	Sorcs2
Mgst1	Aldh1l1	Fam198b	Nceh1	Adcy7	Tmcc3
Lamc2	Hsd17b7	Aldh1l2	Prnp	Lgmn	Arl2bp
Smarcd3	Cadm4	Efnb2	Cldn4	Kif3c	Vamp5
Fam114a1	Parp3	Gbp4	Mapre3	Capns1	Ptrf
Lmna	Cnn2	Slc25a24	Eml2	Gng5	Sdsl
Lrp1	Myof	Dpysl3	Scrn1	Sash1	Pafah1b3
Abcg2	Rhod	Calu	Osbpl5	Pkp2	Creld1
Ttc39c	Myadm	Cnp	Ocrl	Stom	Capg
Plec	Cd109	Tmem63a	Ctbs	Rhoc	Rgs12
Inpp1	Ampd3	Gdi1	Map6	Tmprss11e	Lpp
Samd9l	Cast	Cd47	Acss2	Pdlim2	Filip1l
Dnajb2	Synm	Cdh19	Cenpu	Rab3a	Pak1

Table 4 - TG2 KO 2 vs WT Proteomics upregulated targets:

Cp	S100a11	Fam129a	Serpinb9b	P3h2	Epb41l3
Map2	Cald1	S100a10	Acsbg1	Mbp	Akap12
Anxa5	Adssl1	Gbp2	Fam107b	Dpysl3	Tagln2
Anxa2	Hoxb9	Plekho1	Pdlim2	Pdk1	Steap1
Filip1l	Myof	Ecm1	L1cam	Il1rap	Synm
Tppp3	Tpm4	Fam198b	Cyr61	Ak1	Ifi47
Itga6	Ctsh	Gng5	Fosl1	Cldn4	F3
Synpo	Sphk1	Ehd2	Anxa1	Fn1	Cxadr
Anxa3	Cav1	Mpp2	Postn	Abcg2	Alcam
Lgmn	Nceh1	Prkcq	Dab2ip	Nexn	Sparc
Prnp	Aldh1l1	Parva	Rdh10	Ctsb	Lmna
Rgs12	Pls3	Jun	Fndc3b	Podxl	Prkci
Emb	Pkp2	Notch2	Rhobtb3	Serpinb9	Espn
Rcn2	Tmcc3	Parp3	Pls1	Dnajb2	Ak4
Smap	Nfix	Tst	Gbp4	Sun2	Hdgf
Vamp5	Macc1	Tns1	S100a4	Creld1	Jmy
Rhoc	Ahnak	Steap2	Slc27a1	Samd9l	Zyx
Ppm1l	Myl6b	Vcl	Mcam	Anxa7	Pcbd2
Nes	Rbm20	Sdcbp2	Adamts1	Plod2	Itga5
Iqgap3	Myl12a	Myl6	Lsr	Gramd4	Snap47
Ttc39c	Pip5k1a	Polk	Cnn2	Serpinb6a|Serpinb6	Gpc6
Cnn3	Cobll1	Trim16	Rhod	Samd4a	Gxylt2
Cpe	Aox1	Actn1	Snx16	Smarcd3	Cadm1
Cd109	Pdlim7	Pdlim1	Cdk18	Cast	Cnp
Cd47	Dnajb4	Sci	Flna	Plec	Rab25
Upp1	Jund	Ugdh	Cdc42ep1	Phykp	Npepl1
Rras	Inpp1	Palm2	Magi3	Dlg3	Hnrnph3
Ccnd1	Cst3	Afmid			
