# Supplementary figures and images for: Transglutaminase Type 2-MITF axis regulates phenotype switching in skin cutaneous melanoma
Source: Cell Death Dis. 2023 Oct 28;14(10):704. doi: 10.1038/s41419-023-06223-y (PMC10613311; doi:10.1038/s41419-023-06223-y)

Fig. 2b

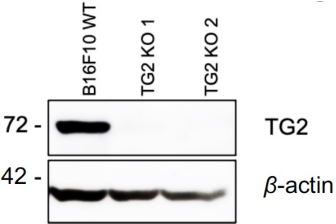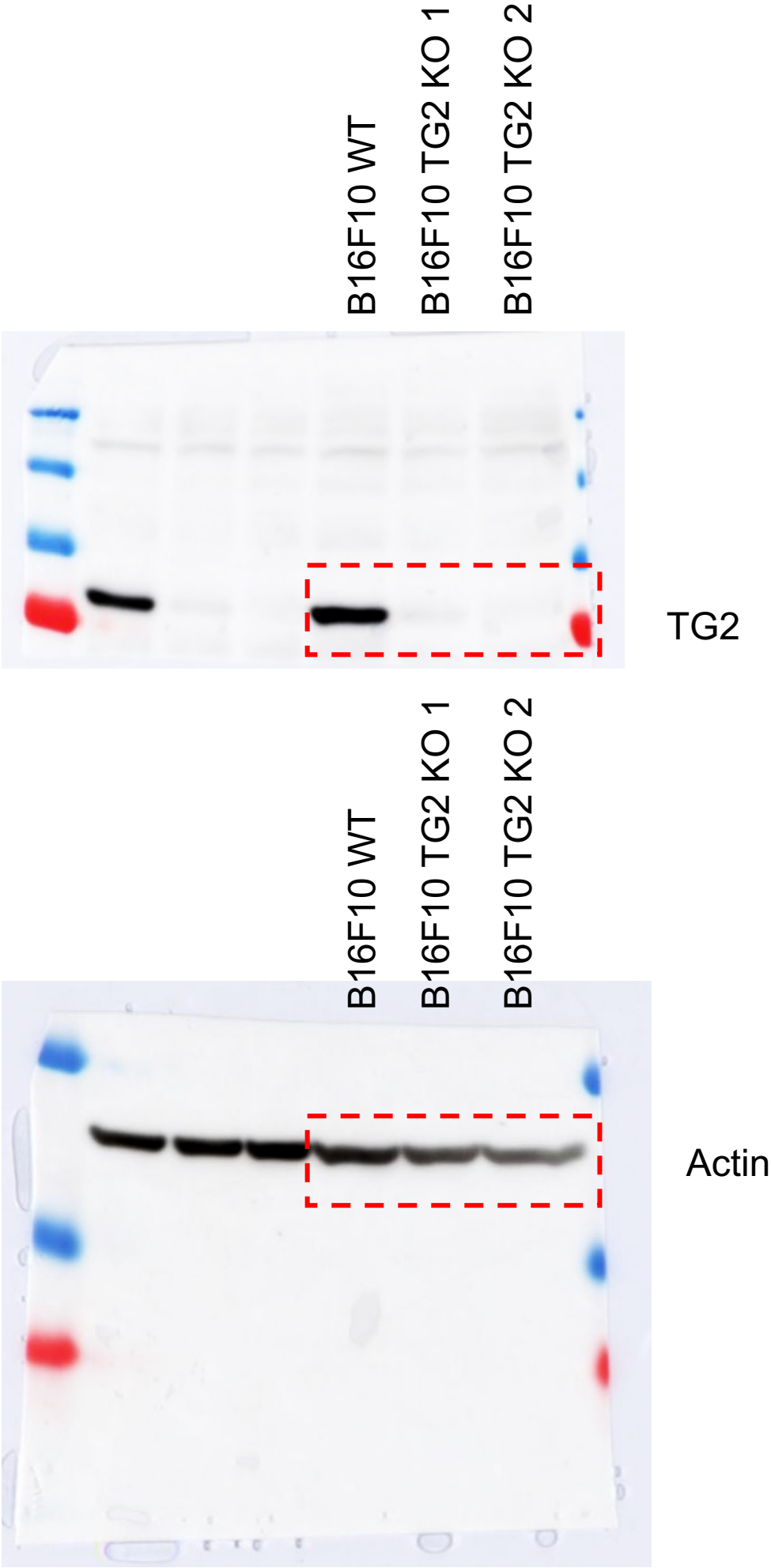

Fig. 3f

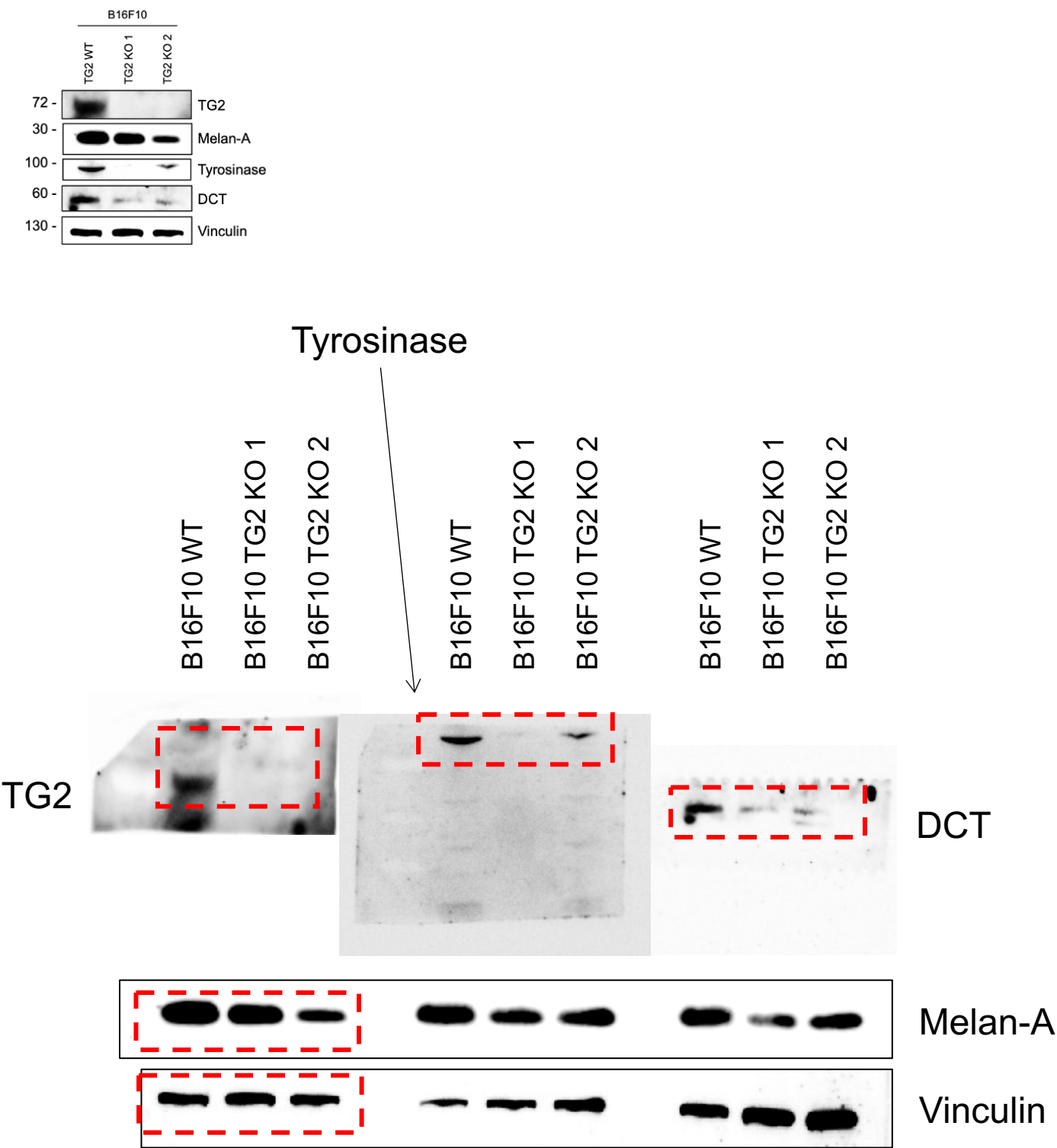

Fig. 3g

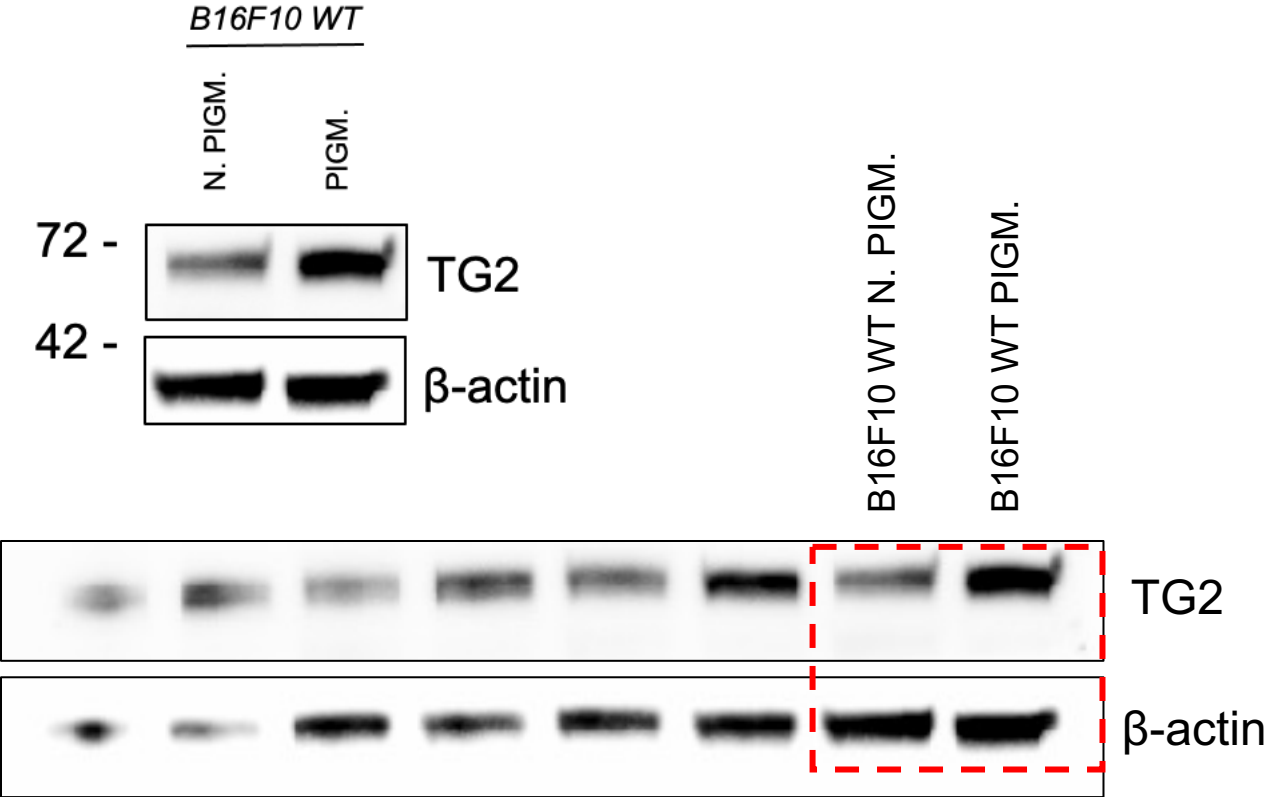

Fig. 4a

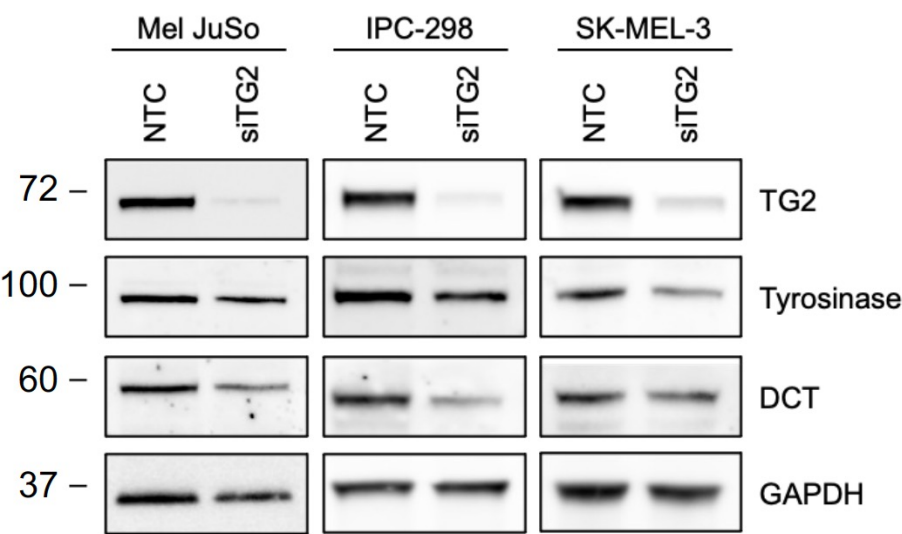

Mel JuSo

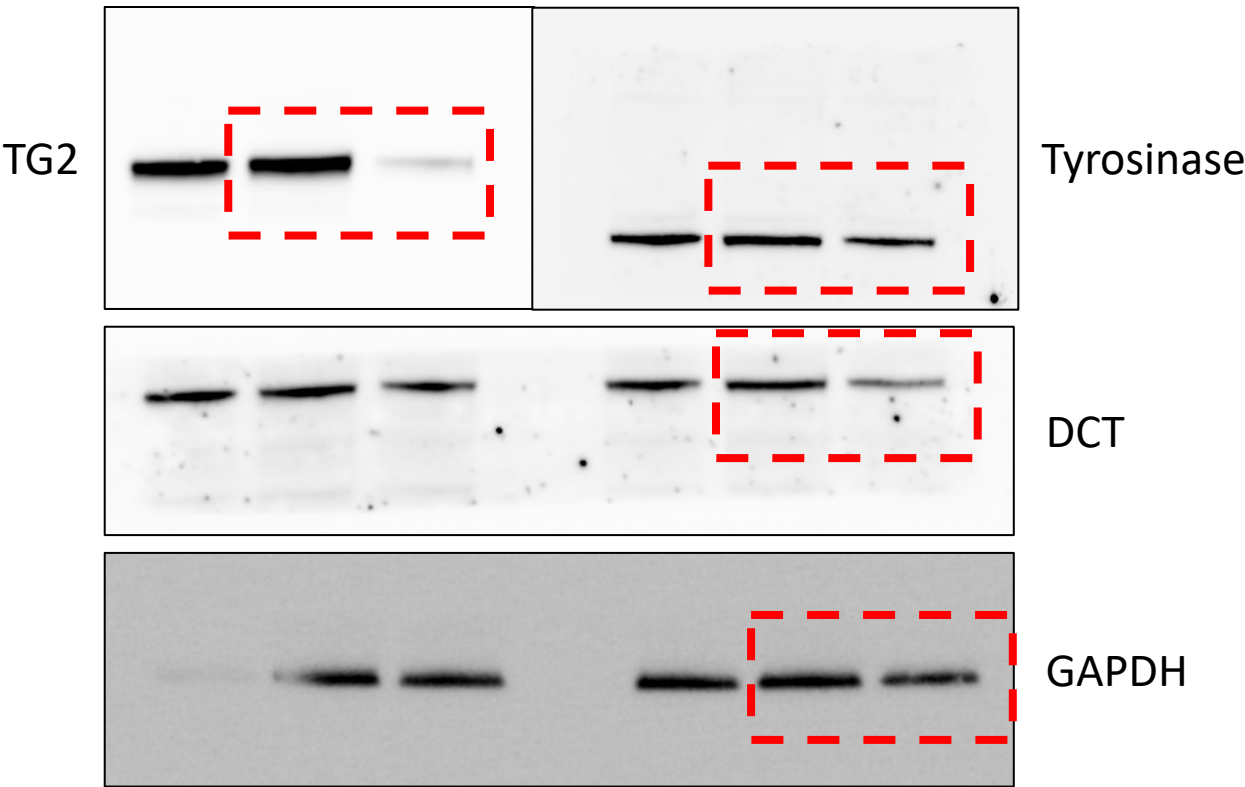

Fig. 4a

IPC-298

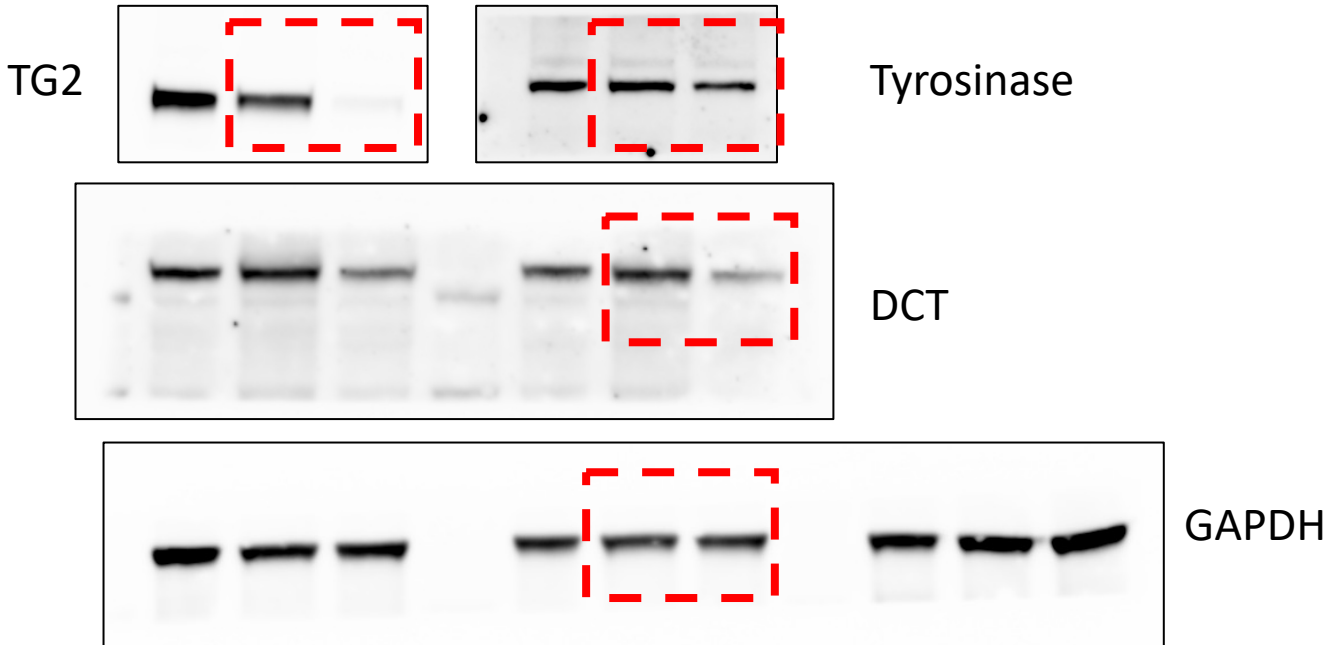

SK-MEL-3

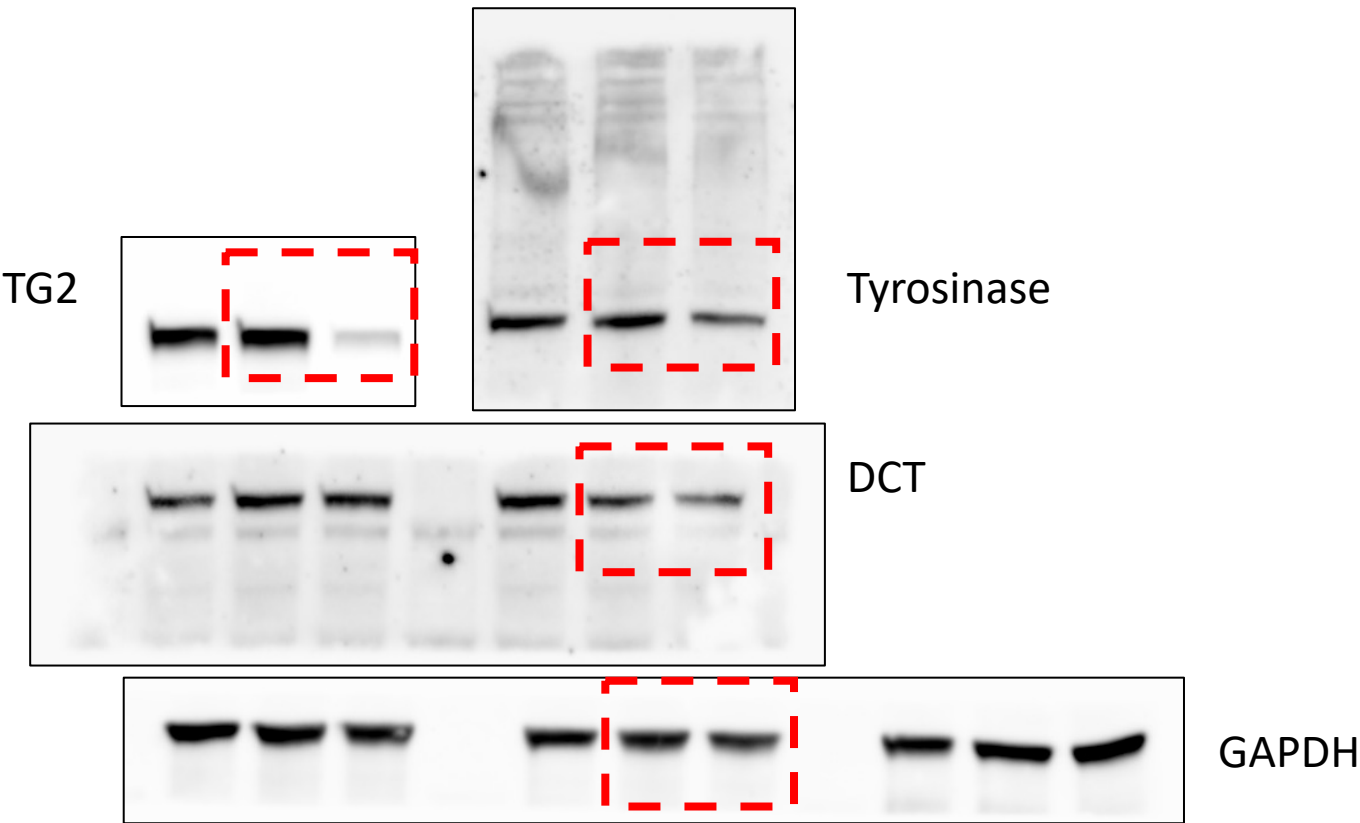

Fig. 5c

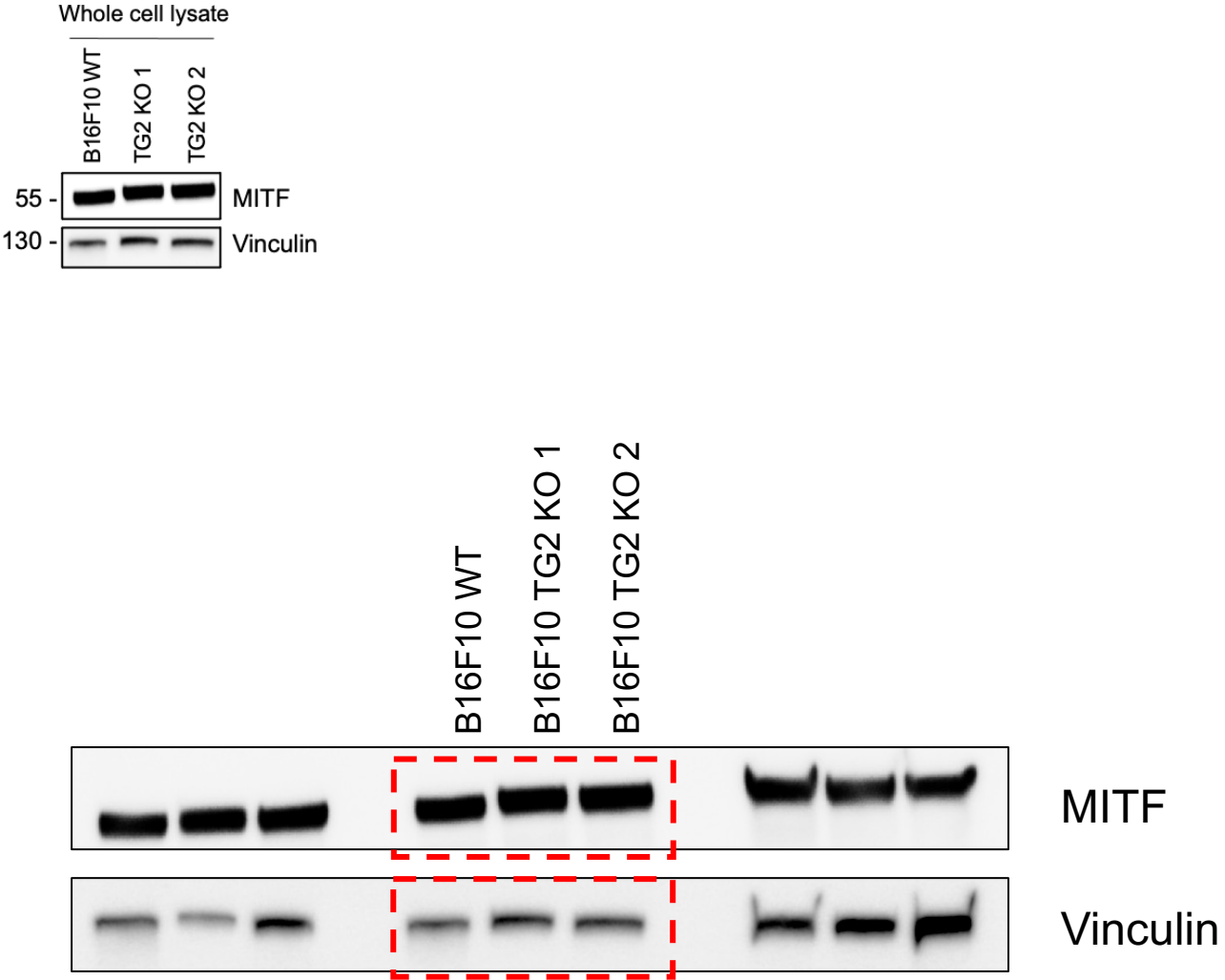

Fig. 5d

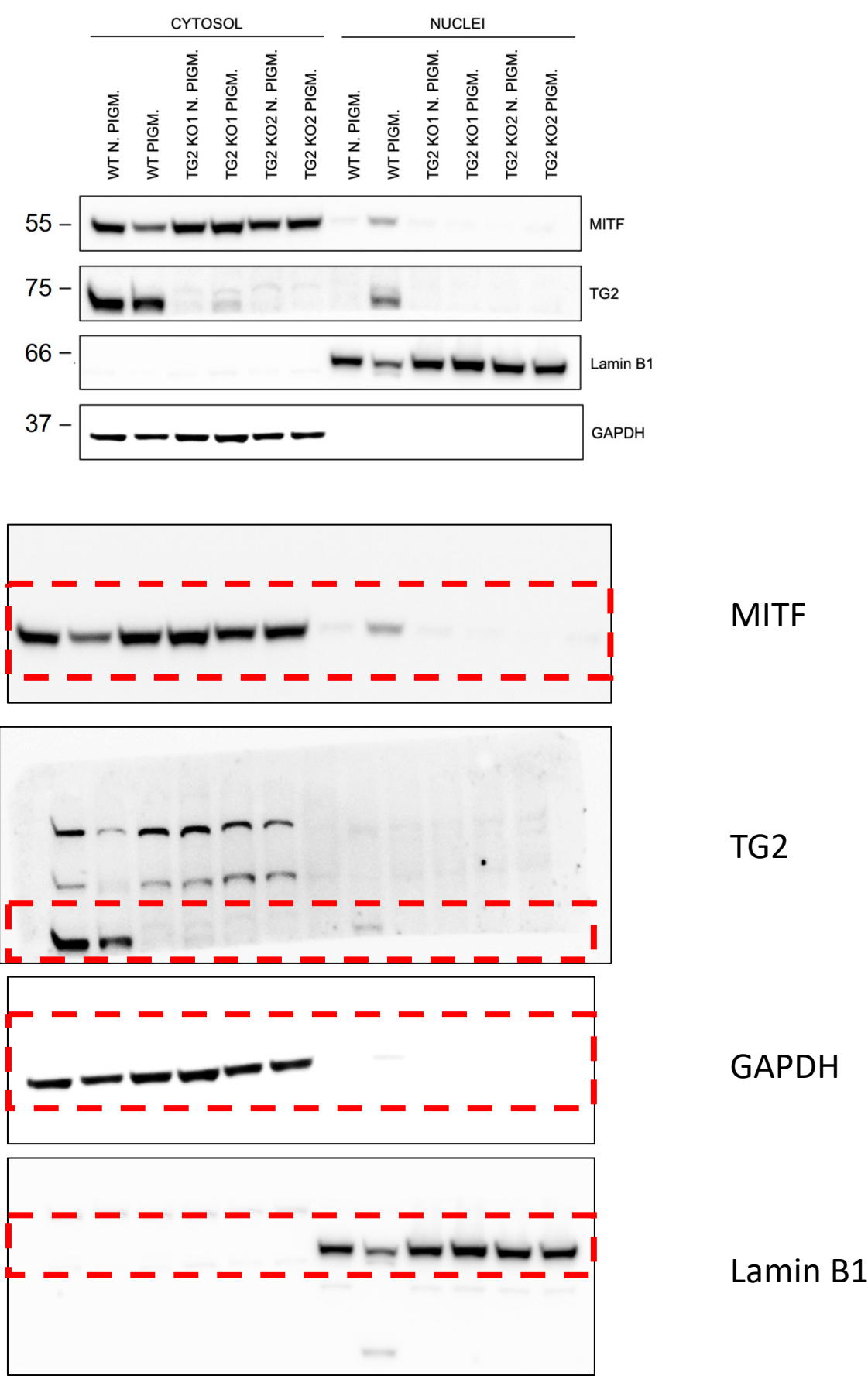

Fig. S3J

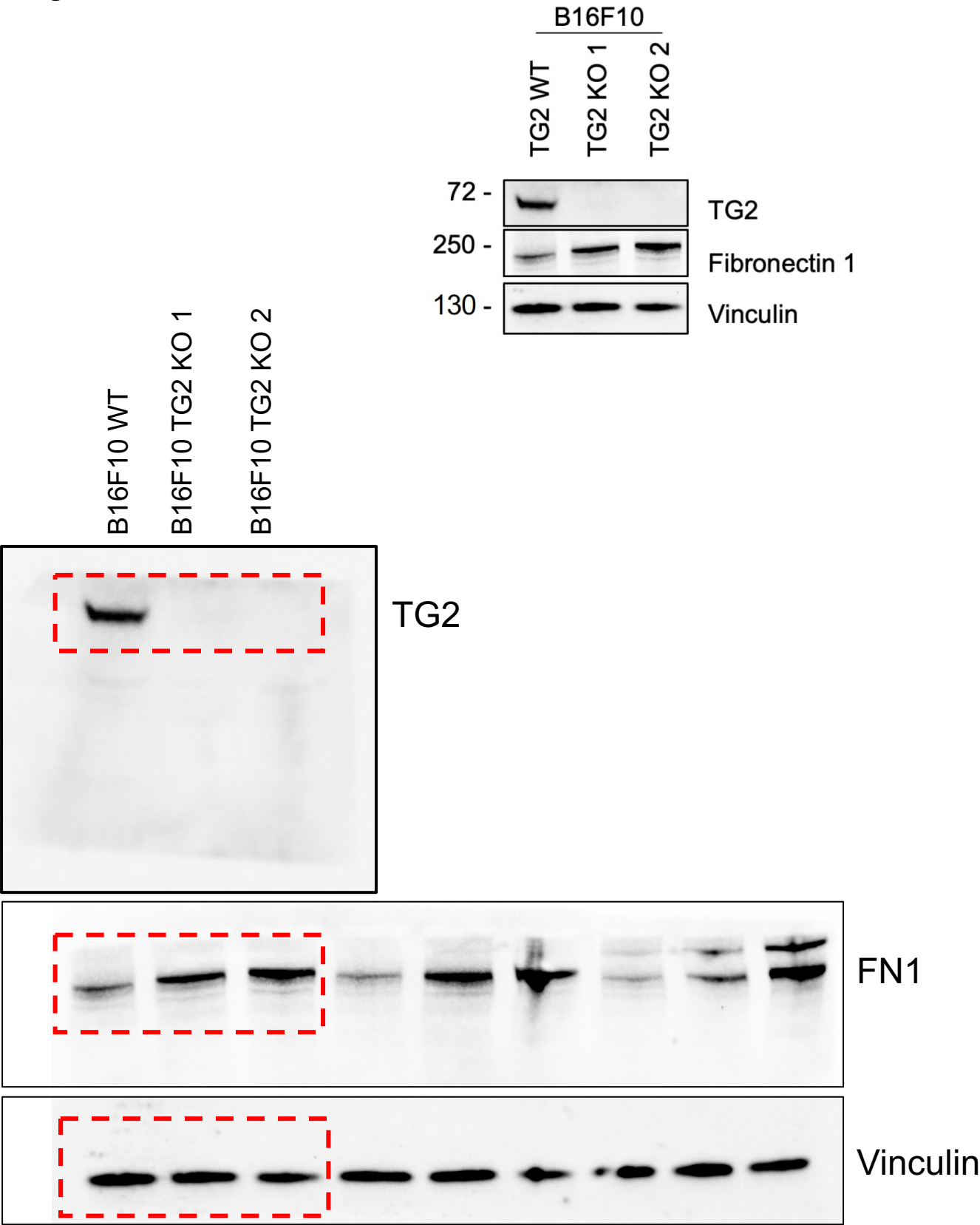

Fig. S6c

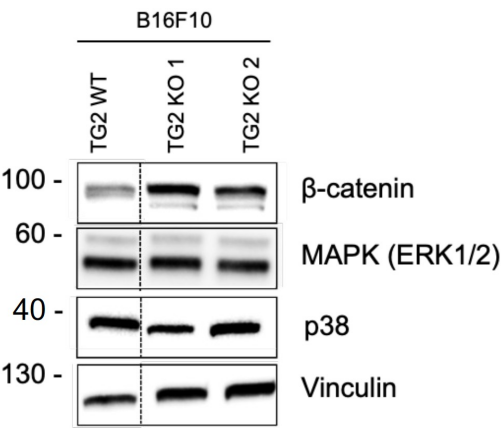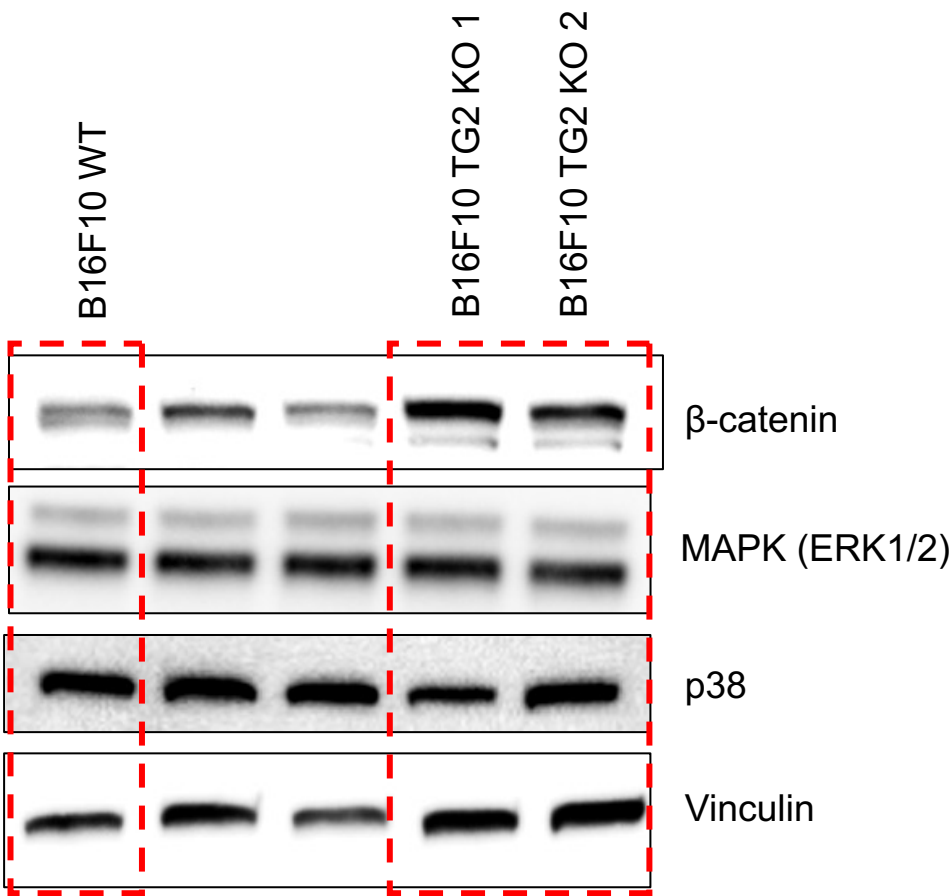

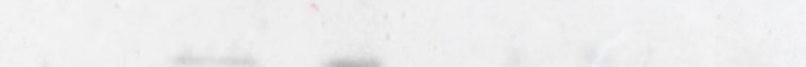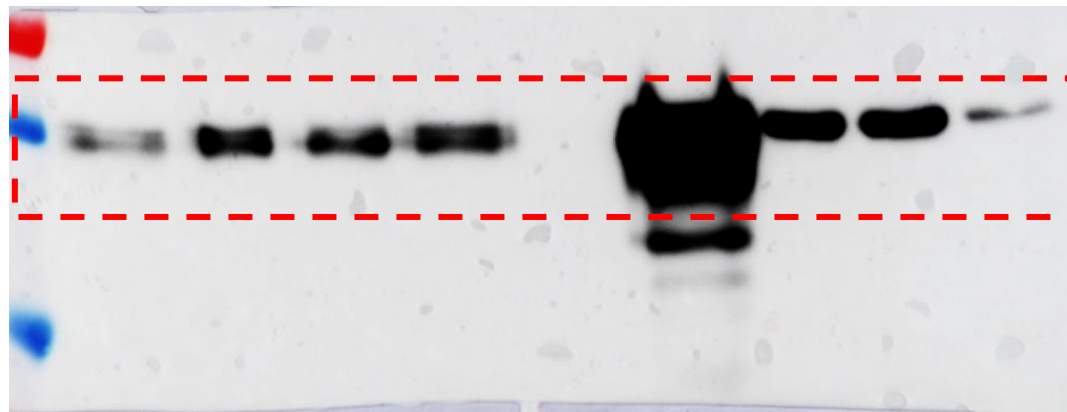

Supplement: Supplementary file 2 — Original Data File [file 41419_2023_6223_MOESM2_ESM.pdf]
